# Effects of different exercise interventions on quality of life in breast cancer survivors after treatment: a systematic review and network meta-analysis

**DOI:** 10.3389/fonc.2026.1775358

**Published:** 2026-04-30

**Authors:** Xiangyu Zhao, Yuanxing Wang, Yanbo Yi, Haitao Zhan, Hao Chen, Qiao Song

**Affiliations:** 1 Department of Sports Education, Zibo Normal College, Shandong, Zibo, China; 2 Police Physical Education Research Department, Zhengzhou Police University, Henan, Zhengzhou, China; 3 Department of Global Management, KyungHee University, Suwan, Republic of Korea; 4 School of Sports Education, Yantai University, Shandong, Yantai, China; 5 School of Sports Management, Dongshin University, Naju, Republic of Korea

**Keywords:** breast cancer survivors, combined exercise, exercise intervention, network meta-analysis, quality of life

## Abstract

**Background:**

With more than 7.8 million breast cancer survivors worldwide and five-year survival rates approaching 90%, optimizing quality of life (QoL) has become a central priority in survivorship care. Exercise is a cornerstone non-pharmacological intervention; however, current guidelines lack modality-specific evidence, and existing meta-analyses often rely on overly broad classifications that obscure clinically meaningful differences among exercise types.

**Methods:**

This systematic review involved comprehensive searches of six electronic databases (Embase, PubMed, Web of Science, the Cochrane Library, EBSCO, and Scopus) from inception through October 2025. We identified randomized controlled trials (RCTs) that evaluated how exercise interventions affect QoL in breast cancer survivors. Using frequentist network meta-analysis, we synthesized the evidence to determine which exercise modalities demonstrated superior effectiveness across the included RCTs.

**Results:**

Sixty-nine randomized controlled trials involving 5,294 breast cancer survivors from 19 countries met the inclusion criteria. All exercise modalities except high-intensity interval training and Pilates were associated with significant improvements in QoL compared with usual care. Surface under the cumulative ranking curve analyses identified combined exercise as the most effective modality (SMD = 1.40, 95% CI: 0.95–1.86; SUCRA = 95.4%), followed by Tai Chi (SMD = 0.98, SUCRA = 71.5%), aerobic exercise (SMD = 0.79, SUCRA = 63.4%), Qigong (SMD = 0.77, SUCRA = 60.1%), yoga (SMD = 0.58, SUCRA = 45.4%), and resistance training (SMD = 0.54, SUCRA = 42.1%).

**Conclusions:**

Combined exercise integrating aerobic and resistance components provides the greatest improvement in QoL among breast cancer survivors. These findings offer hierarchical, modality-specific evidence to support personalized exercise prescription in survivorship care.

**Systematic review registration:**

https://www.crd.york.ac.uk/PROSPERO/view/CRD420251173721, identifier CRD420251173721.

## 1 Introduction

Breast cancer is the most prevalent malignancy worldwide, with approximately 2.3 million new diagnoses annually (1). Advances in early detection and multimodal therapy have led to five year survival rates approaching 90% for early stage disease (2), transforming breast cancer from an acute life-threatening illness into a chronic condition requiring prolonged survivorship care. This epidemiological transition has produced an unprecedented survivor population, exceeding 4.3 million in the United States alone and projected to reach 5.3 million by 2035 (3), while globally more than 7.8 million women are living within five years of diagnosis (1). As survival rates continue to improve, the focus of oncology care has progressively expanded beyond tumor control to address the complex and multifaceted challenges of survivorship, thereby positioning quality of life (QoL) optimization as a central priority for this growing population.

Completion of primary cancer treatment often marks the beginning rather than the resolution of health challenges for breast cancer survivors. Life-saving multimodal therapies frequently produce persistent, multidimensional sequelae that substantially diminish QoL (4). Physical impairments commonly affect multiple systems, including reduced cardiorespiratory fitness, muscle weakness, and diminished bone density. Treatment-related symptoms such as chronic fatigue, pain, lymphedema, and peripheral neuropathy further compromise functional capacity and physical well-being (5, 6). Psychological distress remains highly prevalent, with anxiety, depression, and fear of cancer recurrence persisting for years after treatment in many survivors, thereby substantially compromising emotional QoL (7). Cognitive and social domains are likewise disrupted, manifesting as executive dysfunction, altered body image, sexual dysfunction, and reduced occupational capacity, all of which further diminish overall QoL (8–11). These sequelae interact synergistically, producing cascading effects that perpetuate disability, psychological distress, and progressive QoL deterioration. The chronic and multifactorial nature of QoL impairment in post-treatment survivors underscores the urgent need for evidence-based rehabilitation strategies capable of addressing these holistic needs (3).

Exercise has emerged as a cornerstone non-pharmacological intervention for improving QoL in breast cancer survivors (BCS), supported by robust mechanistic and clinical evidence. Regular physical activity induces favorable physiological adaptations, including enhanced cardiorespiratory capacity, preservation of lean mass, improved bone density, and attenuation of systemic inflammation through downregulation of proinflammatory cytokines (12, 13). Concurrently, exercise confers psychological benefits, improving self efficacy, reducing emotional distress, and fostering resilience (14, 15). These multidimensional improvements collectively contribute to enhanced overall QoL, a comprehensive outcome that captures the holistic impact of exercise interventions across physical, emotional, and social domains. Recognition of these benefits has prompted the incorporation of structured exercise into clinical guidelines. ASCO (American Society of Clinical Oncology)recommends weekly participation in moderate intensity aerobic activities totaling at least 150 minutes accompanied by twice weekly resistance training (16), while ACSM (American College of Sports Medicine) endorses supervised exercise programs as safe, feasible, and effective for functional restoration (17).

Contemporary exercise oncology encompasses a broad spectrum of intervention modalities, each providing distinct physiological stimuli and theoretical advantages for enhancing QoL. Aerobic exercise, such as walking, cycling, and swimming, primarily enhances cardiovascular efficiency and metabolic health, thereby improving physical functioning dimensions of QoL (18, 19). Resistance training, based on progressive overload principles, focuses on improving muscular strength, which directly contributes to functional independence and physical wellbeing (20, 21). Mind body practices, including yoga, Tai Chi, and Qigong, integrate physical movement with meditative components, potentially offering enhanced psychological QoL benefits through stress reduction and emotional regulation (22, 23). High-intensity interval training promotes rapid cardiovascular adaptation, whereas Pilates targets physical limitations while simultaneously supporting social, emotional, and mental wellbeing (24–26). Combined interventions integrating aerobic and resistance components may yield synergistic benefits across multiple QoL domains (27, 28). Despite this diversity, comparative effectiveness across modalities for improving overall QoL remains insufficiently understood. Given that QoL represents the most comprehensive and clinically meaningful outcome for evaluating survivorship interventions establishing which exercise modalities most effectively enhance QoL constitutes a critical research priority.

Although multiple exercise modalities have demonstrated potential for enhancing QoL, existing meta-analyses exhibit methodological limitations that constrain clinical applicability. A systematic review of recent network meta-analyses (29, 30) reveals persistent gaps in modality-specific evidence. Han et al. (2024) grouped all mind-body practices into a single undifferentiated category, obscuring potential differences among yoga, Tai Chi, Qigong, and Pilates (30). Li et al. (2024) advanced the field by separating yoga from other mind-body modalities, yet still combined Tai Chi, Qigong, and Pilates into one node (29). Furthermore, both reviews included mixed populations comprising patients undergoing active treatment alongside post-treatment survivors, conflating groups with fundamentally different QoL challenges and rehabilitation needs. These survivors experience distinct patterns of QoL deterioration requiring targeted rehabilitation strategies different from those appropriate during active treatment (31). Emerging exercise methods, such as high-intensity interval training, have not yet been studied as independent intervention nodes in published network meta-analyses. These limitations impede precise identification of optimal exercise prescriptions for improving QoL in specific survivor populations.

Network meta-analysis (NMA) offers a rigorous analytical framework capable of simultaneously comparing multiple interventions through the synthesis of direct and indirect evidence (32, 33). This approach enables the generation of comprehensive rankings of intervention effectiveness, thereby providing clinicians with actionable guidance for exercise prescription. Accordingly, this study aims to systematically evaluate the effects of specific exercise modalities on the quality of life of post-treatment BCS, establish comparative effectiveness rankings, and produce evidence-based recommendations for optimizing exercise prescription in BCS. By addressing critical gaps in existing research, this study supports the development of personalized, targeted rehabilitation strategies for this growing population.

## 2 Methods

This systematic review incorporating network meta-analysis adhered to the Preferred Reporting Items for Systematic Reviews and Meta-Analyses (PRISMA) statement, with additional compliance to the NMA extension outlined by Hutton, Salanti (34). We prospectively registered the study protocol in the International Prospective Register of Systematic Reviews (PROSPERO) under registration number CRD420251173721.

### 2.1 Data sources and search strategy

We performed a comprehensive search of six electronic databases: PubMed, Web of Science, Embase, the Cochrane Library, EBSCO, and Scopus, covering all available records up to October 2025. Our search approach integrated both controlled vocabulary (including Medical Subject Headings) and free text keywords addressing breast cancer, physical activity interventions, and quality of life measures. Using the PubMed strategy as our template, we incorporated terminology related to breast malignancies, patients and survivors, various physical activity types (including aerobic exercise, resistance training, combined modalities, high-intensity interval training, Pilates, yoga, Qigong, and Tai Chi), quality of life assessment parameters, and randomized controlled trial methodology. Database specific search syntaxes were tailored to align with individual indexing systems and search algorithms. We imposed no language limitations on our searches. Additionally, we manually examined reference lists from eligible studies and pertinent systematic reviews to ensure comprehensive study capture. Detailed search strategies for each database appear in **Supplementary Appendix 1**.

### 2.2 Inclusion and exclusion criteria

Eligibility criteria for study inclusion were as follows: (1) Population: Adult survivors (≥18 years) with histologically confirmed breast cancer who had completed curative therapy, including surgery, systemic chemotherapy, radiotherapy, or combinations of these treatments, predominantly restricted to those with early to locally advanced stage disease. (2) Intervention: Enrollment in a supervised physical activity program lasting at least four weeks. Acceptable exercise formats comprised aerobic training, resistance training, combined aerobic–resistance regimens, high-intensity interval training (HIIT), yoga, Pilates, Tai Chi, and Qigong, with comprehensive classifications detailed in **Supplementary Appendix 2**; (3) Comparator: Standard care, waitlist control groups, or other exercise based interventions; (4) Outcomes: Quality of life measured through psychometrically validated tools, including the EORTC QLQ-C30, SF-36, FACT-B, EuroQol-5D, FACT-G, or equivalent standardized instruments (an exhaustive list of measurement tools appears in **Supplementary Appendix 3**); and (5) Study design: Randomized controlled trials (RCTs).

The below exclusion criteria were applied: (1) were conference abstracts, letters, reviews, protocols, or other nonprimary research; (2) were duplicate publications or multiple reports from the same dataset; (3) included participants undergoing active treatment (chemotherapy or radiotherapy),; (4) included patients with cancers other than breast cancer or mixed cancer cohorts without separable breast cancer data; (5) combined exercise with additional interventions that could not be isolated (e.g., dietary modification, psychological therapy); (6) lacked sufficient data for quantitative synthesis and no clarification could be obtained from study authors; (7) displayed methodological flaws or poor quality judged likely to bias results; (8) did not provide full-text availability.

### 2.3 Study selection and data extraction

Two independent reviewers (XY and YX) assessed all identified records by examining titles and abstracts. Full-text articles were then obtained for potentially relevant studies. Any discrepancies during the selection process were addressed through discussion to reach consensus, with a third reviewer (YB) serving as arbiter in cases where resolution could not be achieved.

A standardized data extraction template, refined through pilot testing, was employed. Two reviewers independently extracted predefined study-level information, including: (1) study characteristics (first author, publication year, country, sample size, study design, and follow up duration); (2) participant characteristics (mean age, time since diagnosis, and treatment history); (3) Intervention details: modality, session duration, frequency, intensity, total intervention duration, and supervision status; (4) Outcome measures included baseline and post-intervention QoL scores, the specific instruments used, timing of assessments, and extraction of the Global/General quality of life domain from each questionnaire;(5) Adverse events and dropout rates. For multi-arm trials, data from each eligible intervention arm were extracted separately. When studies reported multiple follow up assessments, only immediate post intervention data were included. Authors were contacted when information was missing or unclear. When not directly reported, standard deviations were derived from standard errors, confidence intervals, or T/P values. If these calculations were not feasible, we contacted the study authors by email on at least three occasions to request the missing data.

### 2.4 Risk of bias and quality assessment

We systematically evaluated the methodological quality of included randomized controlled trials using the Cochrane Risk of Bias tool 1.0 (RoB 1.0). The assessment framework encompassed seven key domains: random sequence generation, allocation concealment, participant blinding, outcome assessor blinding, completeness of outcome data, selective reporting of outcomes, and additional bias sources. Each domain received a classification of low, unclear, or high risk of bias. Recognizing the practical challenges inherent to exercise intervention studies, which frequently prevent blinding of participants and study personnel, we did not automatically assign high risk ratings for lack of such blinding. Instead, this determination depended on whether adequate bias reduction measures were employed, including blinded outcome assessment. Two independent reviewers performed the quality assessment. Discrepancies between assessors were reconciled through discussion to establish consensus, with a third reviewer providing adjudication when needed.

### 2.5 Certainty of evidence

The quality of evidence was evaluated using the Grading of Recommendations, Assessment, Development and Evaluations framework. Following its five domains for potential downgrading, namely risk of bias, inconsistency, indirectness, imprecision, and publication bias, we assessed whether the certainty of evidence should be reduced. The overall certainty was then classified as high, moderate, low, or very low (35).

### 2.6 Statistical analysis

We performed pairwise meta-analyses using STATA software (version 18.0). Given that outcomes comprised continuous or ordinal variables measured with diverse instruments, we computed pooled effect sizes as standardized mean differences (SMDs) accompanied by 95% confidence intervals (CIs). We quantified between-study heterogeneity using the I² statistic combined with Cochran’s Q test, where I² values of 25%, 50%, and 75% indicated low, moderate, and substantial heterogeneity, respectively. When heterogeneity was low to moderate (I² ≤ 50%), we employed fixed effects models; conversely, random effects models were utilized when substantial heterogeneity was present. Following recommendations from the Cochrane Handbook, we classified SMDs as small (<0.40), moderate (0.40–0.70), or large (>0.70) effects. We created visual summaries of risk of bias evaluations using Review Manager software (version 5.4).

The NMA was performed within a frequentist framework using Stata software, in line with contemporary methodological recommendations. As all outcomes were continuous and assessed using heterogeneous QoL instruments, treatment effects were synthesized as SMDs with corresponding 95% CIs. The analytical procedure followed a structured, multi-stage approach. The transitivity assumption was first assessed by systematically comparing key clinical and methodological characteristics across studies, specifically participants’ mean age and prior treatment history (e.g., surgery, chemotherapy, and radiotherapy), to ensure the appropriateness and validity of indirect comparisons. A network geometry plot was then constructed, visualizing connections between exercise modalities and controls, with node dimensions reflecting cumulative sample sizes and edge width indicating direct comparison frequency. Consistency of direct versus indirect evidence was assessed via a loop specific interaction model. All pairwise comparison estimates were summarized in league table format. We ranked interventions using mean rank and SUCRA values (0-100%), where higher percentages indicate greater probability of optimal effectiveness for quality of life. Rank probability plots illustrated ranking probability distributions for each intervention. Publication bias and small-study effects were assessed by visually inspecting comparison-adjusted funnel plots for symmetry around pooled estimates.

### 2.7 Sensitivity and subgroup analyses

To test the robustness of our primary results, we performed sensitivity analyses. These analyses included exclusion of studies with low methodological quality (i.e., those rated as high risk of bias). Pooled estimates derived from the full dataset were systematically compared with those obtained from the restricted dataset to determine whether inclusion of lower quality studies influenced the overall results. Findings were considered robust if the direction and statistical significance of effect estimates remained unchanged. All sensitivity analyses utilized SMDs in Stata software (version 18.0).

Additionally, Subgroup analyses were conducted within the network meta-analysis framework to examine the comparative effectiveness of exercise modalities under varying parameter conditions (**Supplementary Appendix 5**). For each parameter stratum containing sufficient network connectivity, separate NMAs were performed to generate stratum-specific effect estimates and SUCRA rankings. This approach enabled comparison of intervention hierarchies across different exercise parameter conditions, thereby identifying whether optimal modality rankings varied by intervention characteristics. Furthermore, following identification of combined exercise as the top-ranked modality in the primary NMA, we conducted additional stratified analyses to examine the effects of CE specifically across the aforementioned parameter categories, providing exploratory guidance on optimal CE prescription parameters. All subgroup NMAs employed random-effects models consistent with the primary analysis.

## 3 Results

### 3.1 Literature search and selection

**Figure 1:**
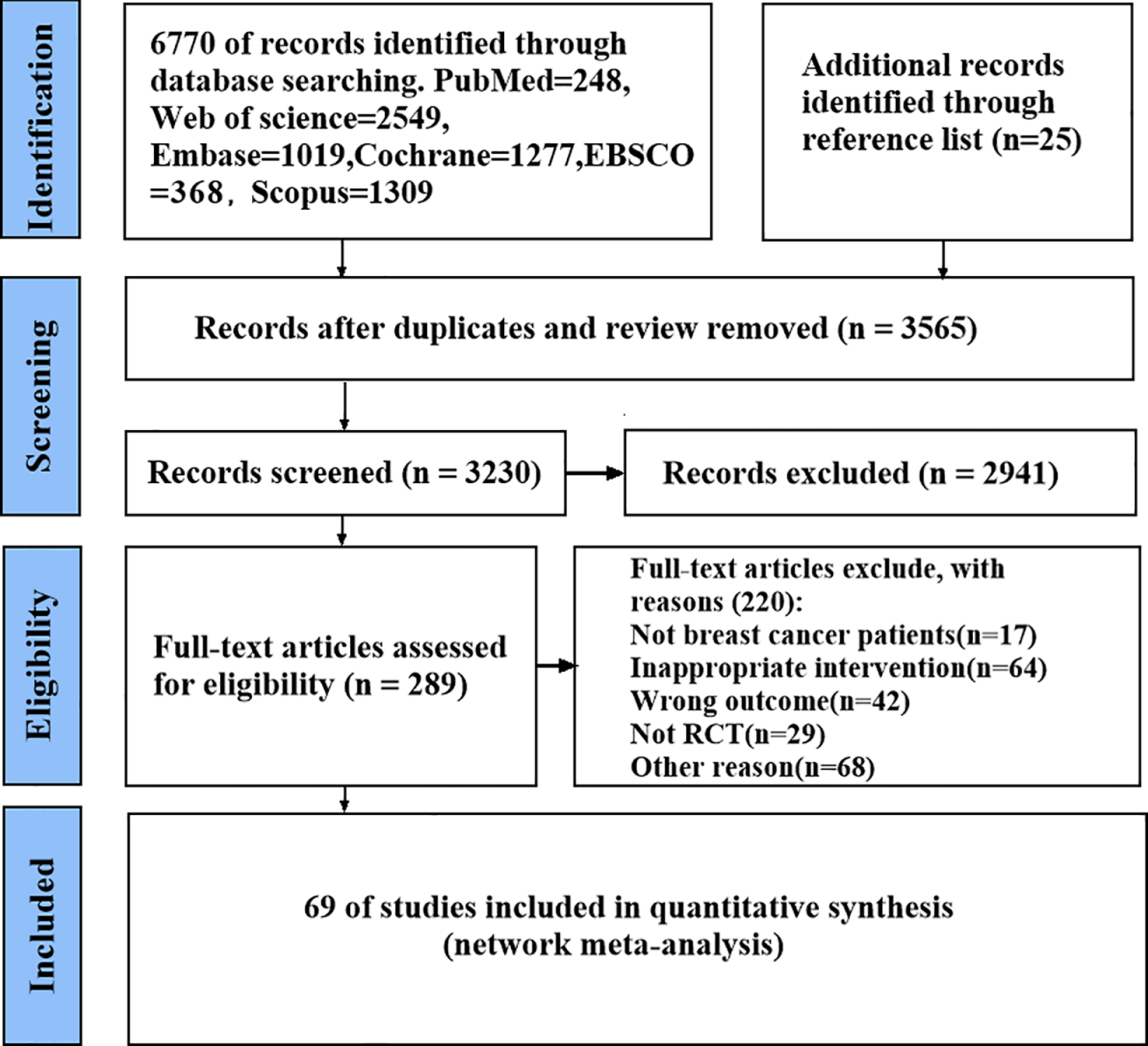
Flowchart of the screening process.

Database searches identified 6,795 records, with an additional 25 citations retrieved through manual screening of reference lists. After removal of duplicates, 3,230 unique records remained for initial screening. Initial screening based on titles and abstracts excluded 2,941 records, leaving 289 articles for full-text evaluation. We excluded 220 of these articles based on the following reasons: non–breast cancer populations (n = 17), interventions inconsistent with protocol specifications (n = 64), outcomes not aligned with study objectives (n = 42), nonrandomized study design (n = 29), and other methodological concerns (n = 68). Ultimately, 69 RCTs met all predefined eligibility criteria and were included in our NMA (6, 7, 12, 14, 19, 20, 24, 26, 28, 36–95) (**Figure 1**).

### 3.2 Study characteristics

The final dataset comprised 69 RCTs involving 5,294 post-treatment BCS, published between 2000 and 2025. The studies demonstrated substantial international representation across 19 countries. Most trials were conducted in the United States (n = 19), while China (n = 8), Canada (n = 6), Australia (n = 5), Spain (n = 4), and Turkey (n = 4) also contributed multiple studies. Brazil, Germany, and Iran each provided three additional studies. Further contributions came from Italy, Latvia, Korea, and the United Kingdom (each n = 2), and single studies from the United Arab Emirates, Netherlands, Serbia, Malaysia, Ukraine, and Denmark. Sample sizes varied notably across trials, ranging from 10 to 160 participants (**Supplementary Appendix 4**).

Aerobic exercise emerged as the most intervention evaluated (n = 21), with combined exercise (n = 14) and resistance training (n = 14) following. Mind-body modalities included yoga (n = 13), Qigong (n = 6), Tai Chi (n = 4), and Pilates (n = 3). High-intensity interval training constituted a smaller subset (n = 3). The timeframe for interventions ranged from 4 to 36 weeks, with 8 weeks representing the typical duration. All included trials utilized supervised exercise formats, with three sessions per week representing the most common frequency pattern.

Quality of life was assessed using a range of validated instruments across the included trials. The most frequently used instrument was the FACT-B (n = 25, 36.2%), followed by the EORTC QLQ-C30 (n = 20, 29.0%), SF-36 (n = 11, 15.9%), FACT-G (n = 7, 10.1%), and FACT-An (n = 2, 2.9%). Other validated instruments, including CARES-SF, EuroQol-5, FACIT-F, and LYMQOL, were each used in 1 study (1.4%). Full details of the quality-of-life instruments used in each study are provided in **Supplementary Appendix 4**.

Breast cancer staging was inconsistently reported, with only 54 of 69 studies (78%) providing any stage distribution data. Of these, most reported mixed-stage cohorts (e.g., Stages 0–III) without sufficient granularity for stratified analysis. Similarly, molecular subtype (ER/PR/HER2 status) was reported in only 3 studies (4%) (**Supplementary Appendix 4**).

### 3.3 Risk of bias assessment

**Figure 2:**
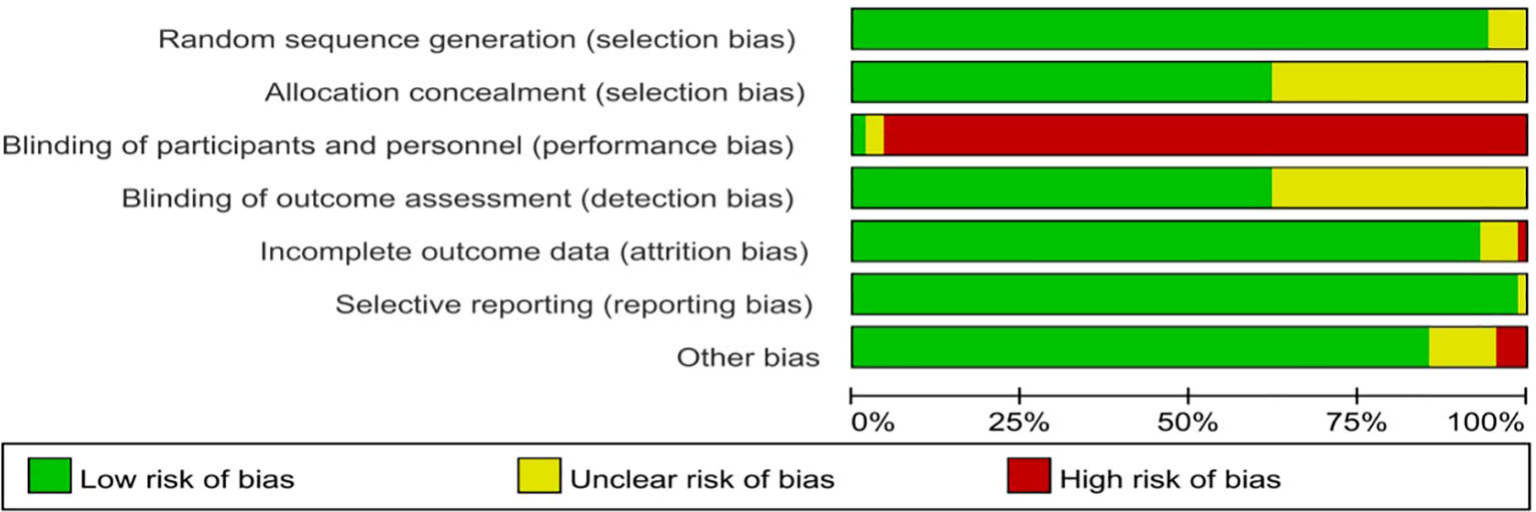
Risk of bias graph for randomized controlled trials.

Methodological quality assessment indicated generally robust study designs across the included trials (**Figure 2**; **Supplementary Appendix 6**). Random sequence generation was adequately described in 94% (n = 65) of studies, with 6% (n = 4) rated as unclear. Allocation concealment was satisfactorily reported in 62% (n = 43), whereas 38% (n = 26) lacked sufficient methodological detail.

Given the inherent challenges of blinding in exercise interventions, performance bias was rated as high in 96% (n = 66) of trials, unclear in 3% (n = 2), and low in only 1% (n = 1). Detection bias demonstrated favorable control: 61% (n = 42) employed blinded outcome assessors, although 39% (n = 27) provided insufficient information. Attrition bias was minimal, with 93% (n = 64) reporting complete outcome data, 6% (n = 4) rated as unclear, and 1% (n = 1) considered high risk. Selective reporting bias was negligible, with 99% (n = 68) classified as low risk and 1% (n = 1) unclear. Other potential sources of bias were generally well-controlled, with 87% (n = 60) rated as low risk, 10% (n = 7) unclear, and 3% (n = 2) high risk.

### 3.4 Pairwise meta analysis and NMA of exercise interventions on QoL

Pairwise meta-analyses were performed first to assess overall exercise intervention effects, followed by NMA for determining the most effective modality. Results from pairwise meta-analysis indicated that exercise interventions significantly enhanced QoL in BCS post-treatment relative to controls (SMD = 0.81, 95% CI: 0.64–0.96), with notable heterogeneity observed (I² = 86.7%; **Supplementary Appendix 7.1**). Loop specific inconsistency analyses indicated that the majority of closed loops did not show statistically significant inconsistency, with confidence intervals generally encompassing zero (P > 0.05). However, an exception was observed in the CON–AE–PILATES loop, which demonstrated a relatively high inconsistency factor (IF = 4.34, 95% CI: 2.30–6.38). This localized inconsistency may reflect the limited number of Pilates trials (n = 3), which increases susceptibility to study-specific outliers, as well as substantial protocol heterogeneity among Pilates studies individual sessions. Additionally, systematic differences in participant characteristics between Pilates and aerobic exercise trials may have violated the transitivity assumption within this loop. Despite this finding, sensitivity analyses excluding Pilates-related comparisons yielded consistent rankings for the remaining modalities, supporting the overall robustness of the network. Despite this localized inconsistency, heterogeneity across most loops remained low (**Supplementary Appendix 7.2**).

**Figure 3:**
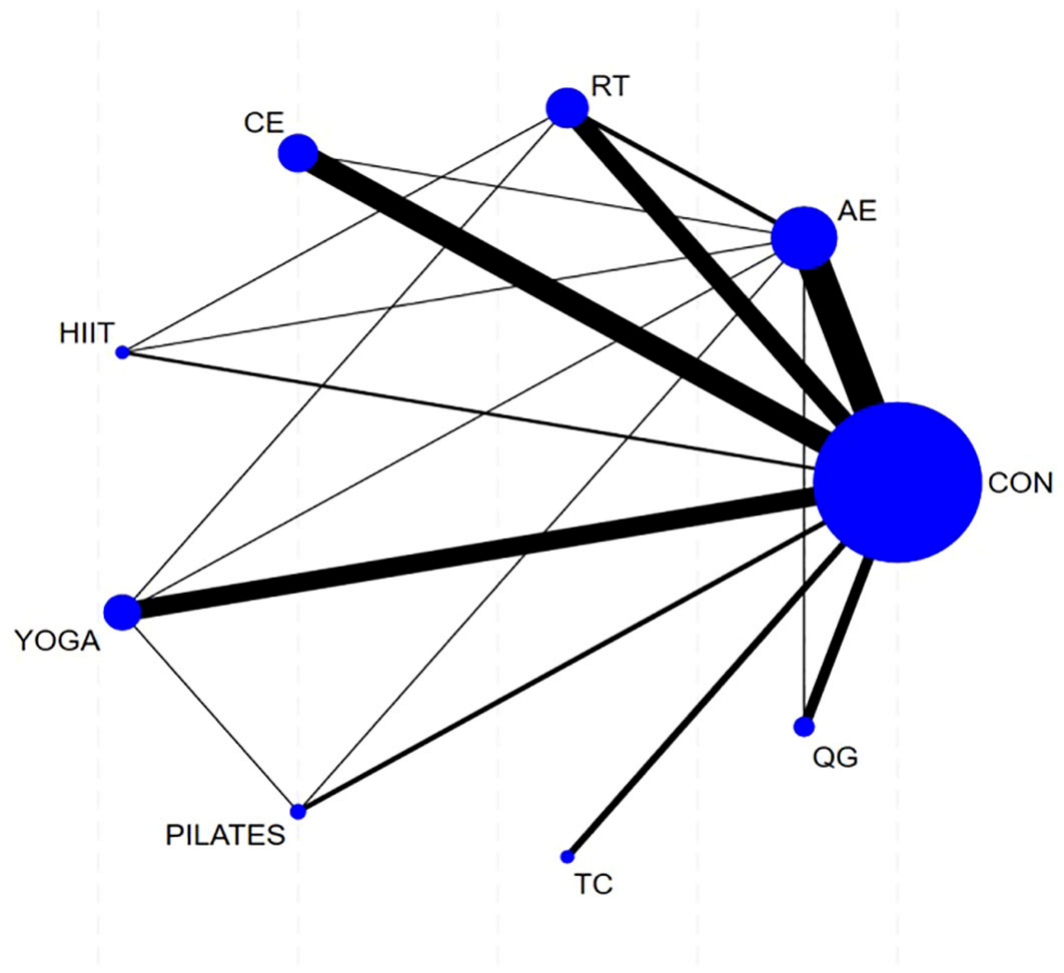
Network plot of comparisons for Qol in the NMA.

The network structure included nine nodes representing eight distinct exercise modalities—aerobic exercise, resistance training, combined exercise, HIIT, yoga, Pilates, Tai Chi, and Qigong—alongside usual care (**Figure 3**). The dataset primarily consisted of two arm Wtrials (n = 63), supplemented by six three arm trials, resulting in 17 direct comparisons and enabling 19 indirect comparisons across the network. Collectively, these findings support the methodological validity of integrating both direct and indirect evidence in the NMA.

**Figure 4:**
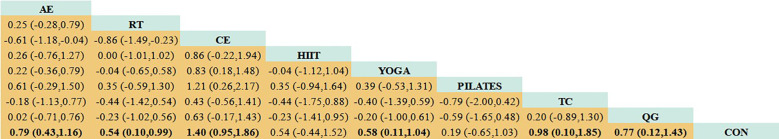
Network meta-analysis matrix of results for QoL.

**Figure 5:**
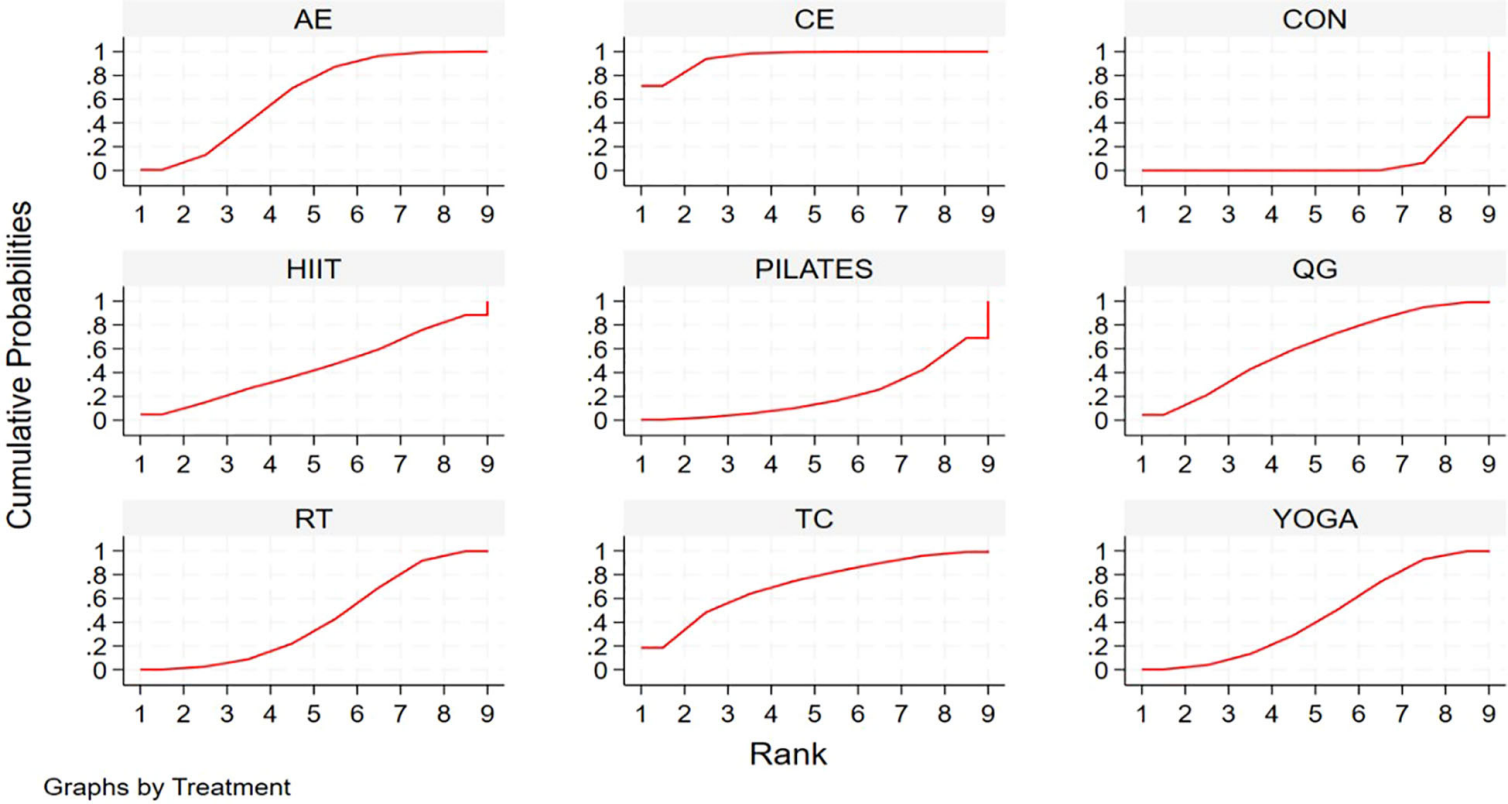
Cumulative probability ranking.

**Figure 4** presents the league table, which summarizes the comparative effects of all evaluated exercise modalities in a matrix of pooled estimates. Corresponding prediction interval plots are provided in **Supplementary Appendix 7.3**. NMA results showed that aerobic exercise (AE), resistance training (RT), combined exercise (CE), yoga, Tai Chi, and Qigong each produced statistically significant quality of life improvements in BCS compared with controls. Effect sizes (SMDs) ranged from 0.54 (95% CI: 0.10–0.99) for RT to 1.40 (95% CI: 0.95–1.86) for CE. In contrast, neither high-intensity interval training (HIIT) nor Pilates showed statistically significant superiority over control interventions. **Figure 5** illustrates the treatment hierarchy derived from cumulative ranking probabilities and SUCRA values. CE showed the highest probability of being most effective, with a 71.2% likelihood of ranking first and a SUCRA value of 95.4% (**Supplementary Appendix 7.4**). Subsequent interventions ranked by effectiveness were: Tai Chi (SMD = 0.98, 95% CI: 0.10–1.85), AE (SMD = 0.79, 95% CI: 0.43–1.16), Qigong (SMD = 0.77, 95% CI: 0.12–1.43), yoga (SMD = 0.58, 95% CI: 0.11–1.04), and RT (SMD = 0.54, 95% CI: 0.10–0.99). The control condition ranked lowest, with a SUCRA value of 6.5%. Furthermore, stratified analyses based on key exercise parameters consistently confirmed the superiority of CE in improving QOL among post-treatment BCS. However, it should be noted that SUCRA values represent probabilistic rankings rather than definitive hierarchies, and overlapping confidence intervals between some modalities indicate uncertainty in relative positioning. Detailed results of these parameter-specific ranking analyses are presented in **Supplementary Appendix 8.1**.

### 3.5 Subgroup analyses

Subgroup analyses stratified by exercise modality indicated that all seven exercise types significantly improved QoL among post-treatment BCS, with the exception of HIIT. Effect sizes (SMDs) ranged from 0.39 for yoga (95% CI: 0.19–0.59) to 1.51 for CE (95% CI: 0.97–2.05) (**Supplementary Appendix 7.1.2**). Subgroup analyses based on exercise parameters examined intervention frequency, session duration, total intervention length, and exercise intensity. Despite variability across subgroups, exercise interventions consistently produced significant enhancements in QoL. When the effects of CE were evaluated across parameter specific subgroups, several noteworthy exploratory findings emerged. CE was associated with the greatest benefits when delivered three or more times weekly, with session durations exceeding 40 minutes, intervention periods longer than 8 weeks, and at a moderate intensity. Given the limited number of CE trials (n = 14) and further subdivision across parameter strata, these comparisons are likely underpowered, and findings require validation in future dedicated trials. Detailed parameter specific subgroup results for CE are provided in **Supplementary Appendix 8.2**. Due to the lack of staging and subtype information, subgroup analysis cannot be performed based on it.

### 3.6 Publication bias assessment and sensitivity analyses

**Figure 6:**
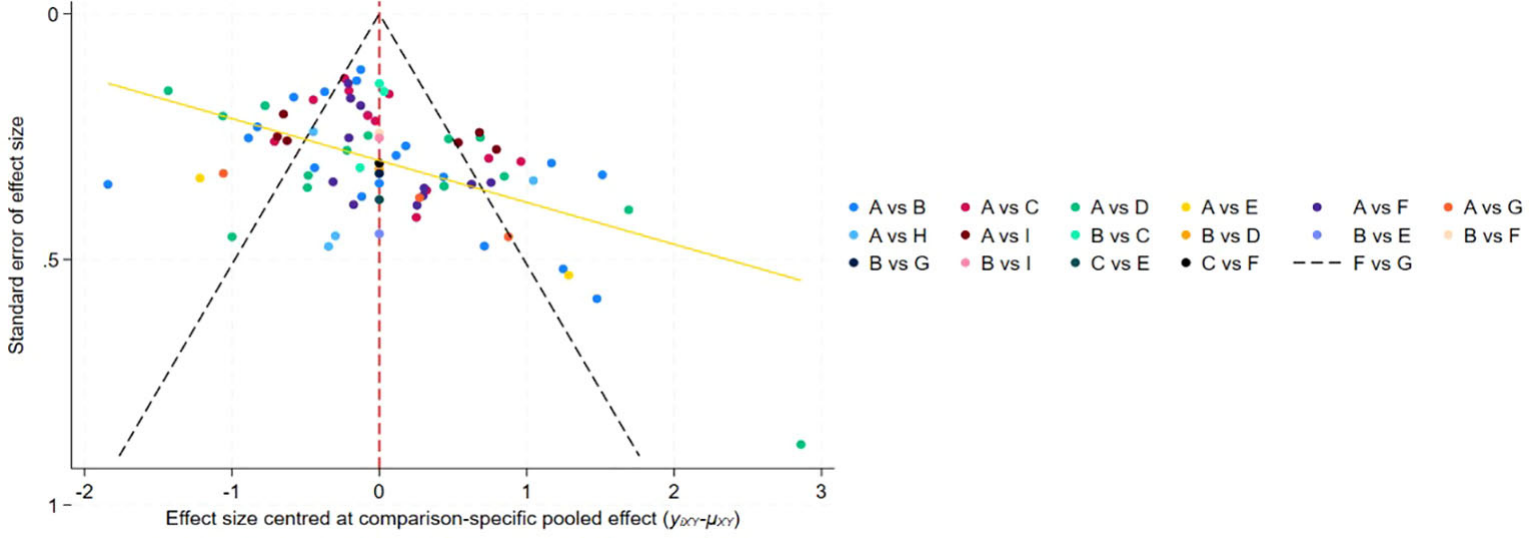
Funnel diagram.

Publication bias was evaluated using comparison adjusted funnel plots (**Figure 6**) and demonstrated acceptable symmetry in the distribution of effect estimates around comparison specific pooled values. The plot showed appropriate dispersion of studies across multiple comparison strata, with effect sizes ranging approximately from –2 to 3 standard deviations. Although a few studies appeared as outliers—particularly within the A vs E comparison (yellow line)—the overall pattern indicated minimal publication bias. Findings from the sensitivity analyses are provided in **Supplementary Appendix 9**. Across all iterations, pooled effect estimates remained closely aligned with the original estimates, with substantial overlap of confidence intervals No single study unduly affected the pooled estimates, indicating that the primary results were stable and robust.

### 3.7 Evidence certainty assessment

The level of evidence for quality of life in this study was rated as low. Detailed results are presented in **Supplementary Appendix 10**.

## 4 Discussion

### 4.1 Overview of main findings

This NMA, encompassing 69 RCTs and 5,294 BCS, provides a comprehensive comparative evaluation of eight distinct exercise modalities on QOL in post-treatment BCS. Previous syntheses have relied on overly simplified classification frameworks that grouped heterogeneous interventions into only four broad categories (29, 30), potentially obscuring meaningful differences between modalities. In contrast, the present study specifically targeted the post-treatment survival phase in BCS, thereby offering more precise and clinically relevant evidence to inform exercise based rehabilitation during this critical period. By simultaneously broadening the range of exercise modalities and restricting the population to a clearly defined survivorship stage, this analysis clarifies the relative effectiveness of specific exercise modalities for QoL outcomes. Pairwise meta analyses confirmed a clear overall benefit from exercise interventions in enhancing QoL. Building on this foundation, the network meta-analysis identified a distinct hierarchical ranking of intervention effects, with combined exercise demonstrating pronounced superiority. CE demonstrated the greatest efficacy, with an effect size of SMD = 1.40 (95% CI: 0.95–1.86) and a SUCRA score of 95.4%, indicating consistent superiority over all other modalities in both effect magnitude and probability-based ranking (low-quality evidence). Furthermore, subgroup analyses focusing on combined exercise delineated exercise parameters associated with the greatest improvements in QOL, providing additional direction for optimizing exercise programming in survivorship care.

### 4.2 Comparative effects of exercise interventions on QoL

The superior efficacy of CE likely reflects synergistic engagement of multiple mechanistic pathways relevant to BCS. At the metabolic and inflammatory level, aerobic exercise reduces circulating pro-inflammatory cytokines including IL-6, TNF-α, and CRP that are implicated in cancer-related fatigue and depression (96), while resistance training attenuates treatment-induced muscle wasting through activation of mTOR-mediated protein synthesis and suppression of myostatin expression (97). These combined anti-inflammatory and anabolic effects may synergistically improve the fatigue and physical functioning domains of QoL. From a neuroendocrine perspective, AE modulates hypothalamic-pituitary-adrenal axis function, reducing the cortisol dysregulation commonly observed (98). From a cardio-oncology perspective, the cardioprotective benefits of aerobic exercise, such as improvements in ejection fraction, reductions in arterial stiffness, and enhanced endothelial function, are especially important in light of the cardiotoxic effects associated with anthracycline-based chemotherapy and trastuzumab commonly used in breast cancer treatment (99). In addition, resistance training contributes to cardiovascular health by improving insulin sensitivity and reducing the prevalence of metabolic syndrome (54). Beyond these physiological mechanisms, exercise enhances psychological factors strongly associated with QoL in BCS, including physical, emotional, social, and functional well-being (54). Studies conducted in diverse survivor populations, such as overweight and ethnically heterogeneous cohorts, consistently report that combined exercise improves quality of life, reduces fatigue and depressive symptoms, and enhances overall physical health status (54, 88). Moreover, the benefits of CE span the survivorship continuum, from early postoperative rehabilitation to long term management of aromatase inhibitor related symptoms (12, 48). These integrated physiological and psychological mechanisms jointly explain the superior efficacy of combined exercise in improving quality of life, with consistent benefits observed across diverse survivor populations and throughout different stages of survivorship. These findings are closely aligned with current international consensus guidelines on exercise for cancer survivors (17).

Our analysis also revealed differential effectiveness among mind body modalities. Tai Chi ranked second, followed by Qigong and yoga. The superior performance of Tai Chi may reflect its integration of low impact movement, controlled breathing, and meditative focus, collectively targeting symptom clusters such as fatigue, sleep disturbance, and depression (42). Comparative studies indicate that Tai Chi produces greater improvements in aerobic capacity, muscular strength, and flexibility than psychosocial support alone (87), alongside favorable changes in neuroendocrine biomarkers including IGF-1 and cortisol (81). Qigong demonstrated consistent benefits for fatigue reduction, sleep quality, and cognitive function, potentially mediated through improvements in anxiety and fatigue (43, 44, 95). Yoga appeared to exert more targeted effects on psychological outcomes, including anxiety reduction and management of menopausal symptoms (14, 70, 76). Such distinctions demonstrate why modality specific categorization is important in exercise oncology research.

AE ranked third in the NMA, consistent with its well-established benefits for cardiorespiratory fitness, functional capacity, and symptom control. Evidence indicates that AE improves global health status and multiple functional domains, including physical, role, and emotional functioning (56, 73). Recent studies incorporating wearable device assisted AE have reported additional benefits for anxiety, depression, fatigue, and sleep disturbance (38). RT, although ranking lowest among interventions demonstrating significant effects, remains essential for restoring muscular strength and body composition—outcomes that may not be fully captured by global QoL instruments (84, 91).

The nonsignificant effects observed for HIIT and Pilates warrant cautious interpretation given the limited number of included trials (n = 3 each). For HIIT, although evidence suggests superior cardiorespiratory adaptations compared with moderate-intensity continuous training (6, 20), increased psychological distress reported in some studies may offset physical benefits when overall QoL is assessed (39). For Pilates, individual trials have reported improvements in functional capacity and psychological wellbeing (24, 85); however, non-specific participation effects have also been noted, indicating that more rigorously controlled trials are required to establish modality specific efficacy (26).

Subgroup analyses focusing on CE yielded several exploratory insights into optimal exercise parameters. CE yielded the most pronounced improvements when prescribed at a minimum frequency of three sessions per week, with individual sessions lasting more than 40 minutes, intervention durations exceeding eight weeks, and delivered at moderate intensity. These findings are broadly consistent with ASCO guidelines of 150 minutes weekly of moderate intensity aerobic activity combined with two weekly resistance training sessions (16). Nonetheless, balancing AE and RT components within CE to achieve optimal duration and frequency for each modality remains challenging and may attenuate potential benefits, as noted previously. Shorter interventions (≤8 weeks) exhibiting larger effect sizes may reflect better adherence to progressive overload principles, whereas longer interventions with fixed parameters may experience diminishing returns and increased attrition. Although exploratory, these findings provide preliminary guidance to inform clinical implementation and future intervention design.

### 4.3 Clinical implications and strengths

These findings carry substantial implications for survivorship care. For clinicians, CE should be prioritized as the first-line exercise prescription for post-treatment BCS seeking comprehensive QoL enhancement. However, CE requires greater time and equipment access, potentially creating barriers for some survivors; thus, prescriptions should be individualized based on patient circumstances, resources, and feasibility. For survivors with specific symptom burdens or preferences, Tai Chi offers an effective alternative, particularly for those experiencing fatigue-sleep-depression symptom clusters. The modality specific rankings enable personalized exercise prescription based on individual needs, functional capacity, and accessibility. Given the high availability and acceptability of exercise interventions, these results can also inform community based self selection of exercise modalities for survivors discharged from clinical care, ultimately supporting the broader goal of implementing structured exercise programs in standard survivorship care.

This study possesses several methodological strengths. It represents the first NMA to employ comprehensive modality specific categorization, distinguishing eight distinct exercise types rather than collapsing interventions into oversimplified categories. The exclusive focus on post-treatment BCS addresses a critical gap, as this population experiences unique and persistent QoL impairments requiring targeted rehabilitation strategies distinct from those appropriate during active treatment. The large dataset spanning 69 RCTs from 19 countries enhances statistical power and external validity. Rigorous consistency testing, sensitivity analyses, and publication bias assessment support the reliability of hierarchical rankings.

### 4.4 Limitations and future research directions

Several limitations of this research warrant consideration. The exclusive inclusion of supervised exercise programs enhances internal validity but limits generalizability to real-world settings, where home-based or unsupervised exercise may be more practical and sustainable for long-term survivors. Performance bias was rated high in most trials because blinding participants to exercise allocation is inherently infeasible. Substantial heterogeneity (I² = 86.7%) likely reflects variability in exercise protocols, such as differences in intervention duration ranging from 4 to 36 weeks, session frequency of 1 to 5 times per week, and training intensity even within the same modality, as well as differences in the sensitivity of QoL instruments and participant characteristics including time since diagnosis, treatment regimens, and baseline functional status, although subgroup analyses identified exercise modality as the primary contributor. Moreover, the lack of consistent staging and molecular subtype data precluded stage-specific analyses, and this unmeasured heterogeneity may confound effect estimates. The limited number of HIIT and Pilates trials constrains the precision of effect estimates for these modalities. In addition, variability in QoL assessment tools may introduce measurement heterogeneity despite all instruments being validated and capturing multidimensional constructs. Finally, the geographic concentration of trials in high-income countries may restrict applicability to resource-limited settings.

Future research should prioritize direct head-to-head comparisons between top-ranked modalities, particularly combined exercise and Tai Chi, to establish comparative effectiveness. Longitudinal follow-up studies are needed to determine the durability of QoL improvements and identify optimal maintenance strategies. Mechanistic investigations incorporating biomarkers, neuroimaging, and psychosocial mediators would help clarify pathways underlying differential modality effects. Additional rigorously designed trials evaluating Pilates are warranted to better define its role in survivorship care. Notably, HIIT did not demonstrate statistically significant benefits in the present analysis and therefore cannot be recommended based on current evidence, especially for survivors with uncertain functional capacity or a history of advanced-stage disease. Expanding research to include diverse and underserved populations will further strengthen equity and enhance the global applicability of exercise oncology findings.

### 4.5 Conclusion

This NMA, synthesizing evidence from 69 RCTs involving 5,294 post-treatment BCS, demonstrates that combined exercise integrating aerobic and resistance training yields the greatest improvements in quality of life (SUCRA = 95.4%), followed by Tai Chi, aerobic exercise, Qigong, yoga, and resistance training. The lack of statistically significant effects observed for HIIT and pilates should be interpreted with caution. These modality specific rankings provide robust hierarchical evidence to inform personalized exercise prescription in survivorship care. Clinicians should prioritize combined exercise as the first line recommendation, while considering individual preferences and symptom profiles when selecting alternative modalities. Future research should explore optimal CE composition and long-term effectiveness.

## Data Availability

The original contributions presented in the study are included in the article/**Supplementary Material**. Further inquiries can be directed to the corresponding author.
