# Supplementary material for: Effects of different exercise interventions on quality of life in breast cancer survivors after treatment: a systematic review and network meta-analysis
Source: Front Oncol. 2026 Apr 30;16:1775358. doi: 10.3389/fonc.2026.1775358 (PMC13195307; doi:10.3389/fonc.2026.1775358)
Supplement: Supplementary file 1 [file Table1.docx]

## Content

[Content 1](#_Toc28143)

[Appendix 1 Search strategy 1](#_Toc7451)

[Appendix 2 The classifications of exercise interventions 7](#_Toc10259)

[Appendix 3 The measurement tools for Quality Of Life outcomes 8](#_Toc5028)

[Appendix 4 Study characteristics 8](#_Toc27060)

[Appendix 5 The Classification of exercise parameters for subgroup analyses 26](#_Toc8875)

[Appendix 6 Risk of bias Summary 28](#_Toc30206)

[Appendix 7 The analysis for outcome 29](#_Toc12879)

[7.1.1 Pairwise analysis for Quality Of Life outcome 29](#_Toc1969)

[7.1.2 Pairwise analysis of quality of life results for different exercises 30](#_Toc8448)

[7.2 Inconsistency test for quality of life 32](#_Toc8032)

[7.3 Predictive interval plot for the quality of life network 33](#_Toc16709)

[7.4 Treatment Relative Ranking of Model 33](#_Toc20662)

[Appendix 8 The analysis for subgroup analysis 34](#_Toc7796)

[8.1 SUCRA curves and league tables under different movement parameters 34](#_Toc28347)

[8.2 subgroup analysis for combined exercise for quality of life 40](#_Toc2132)

[Appendix 9 The results of the sensitivity analysis 44](#_Toc14194)

#

# Appendix 1 Search strategy

Table1 Search strategy in PubMed

| Search strategy |
| --- |
| Search: (((((Breast Neoplasms[MeSH]) OR ((((((((Breast Neoplasm[Title/Abstract]) OR (Breast Cancer[Title/Abstract])) OR (Cancer, Breast[Title/Abstract])) OR (Cancer of Breast[Title/Abstract])) OR (Cancer of the Breast[Title/Abstract])) OR (Breast Carcinoma[Title/Abstract])) OR (Breast Carcinomas[Title/Abstract])) OR (Carcinoma, Breast[Title/Abstract]))) AND (((Survivors[Mesh]) OR (survivor[Title/Abstract])) OR ((Patients[Mesh]) OR (Patient[Title/Abstract])))) AND ((exercise[MeSH]) OR (((((((((((((Exercises[Title/Abstract]) OR (Exercise, Aerobic[Title/Abstract])) OR (Exercise Training[Title/Abstract])) OR (Training, Exercise[Title/Abstract])) OR (resistance training[Title/Abstract])) OR (strength training[Title/Abstract])) OR (combined training[Title/Abstract])) OR (high intensity interval training[Title/Abstract])) OR (Pilates[Title/Abstract])) OR (Yoga[Title/Abstract])) OR (qigong[Title/Abstract])) OR (taichi[Title/Abstract])) OR (taiji[Title/Abstract])))) AND ((Quality of Life[MeSH]) OR ((((Life Quality[Title/Abstract]) OR (Health-Related Quality Of Life[Title/Abstract])) OR (Health Related Quality Of Life[Title/Abstract])) OR (HRQOL[Title/Abstract])))) AND (((randomized controlled trial[Title/Abstract]) OR (randomized[Title/Abstract])) OR (placebo[Title/Abstract])) Sort by: Most Recent |


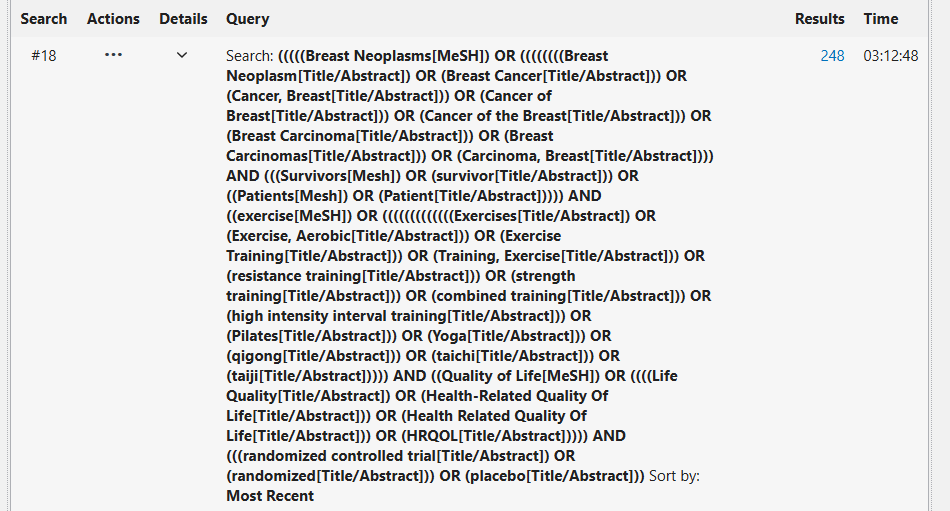


Figure 1 Search strategy in PubMed

Table 2 Search strategy in Embase

| Search strategy |
| --- |
| Search:'cancer, breast':ab,ti or 'breast gland cancer':ab,ti or 'breast gland neoplasm':ab,ti or 'cancer of the breast':ab,ti or 'survivors':ab,ti or 'patients':ab,ti or 'Exercises':ab,ti or 'Exercise, Aerobic':ab,ti or 'Exercise Training':ab,ti or 'Training, Exercise':ab,ti or 'resistance training ':ab,ti or 'strength training':ab,ti or 'combined training':ab,ti or 'high intensity interval training':ab,ti or 'Pilates':ab,ti or 'Yoga':ab,ti or 'qigong':ab,ti or 'taichi ':ab,ti or 'taiji':ab,ti or 'health related quality of life':ab,ti or 'HRQL':ab,ti or 'life quality':ab,ti or 'randomized controlled trial':ab,ti or 'randomized':ab,ti or 'placebo':ab,ti or |


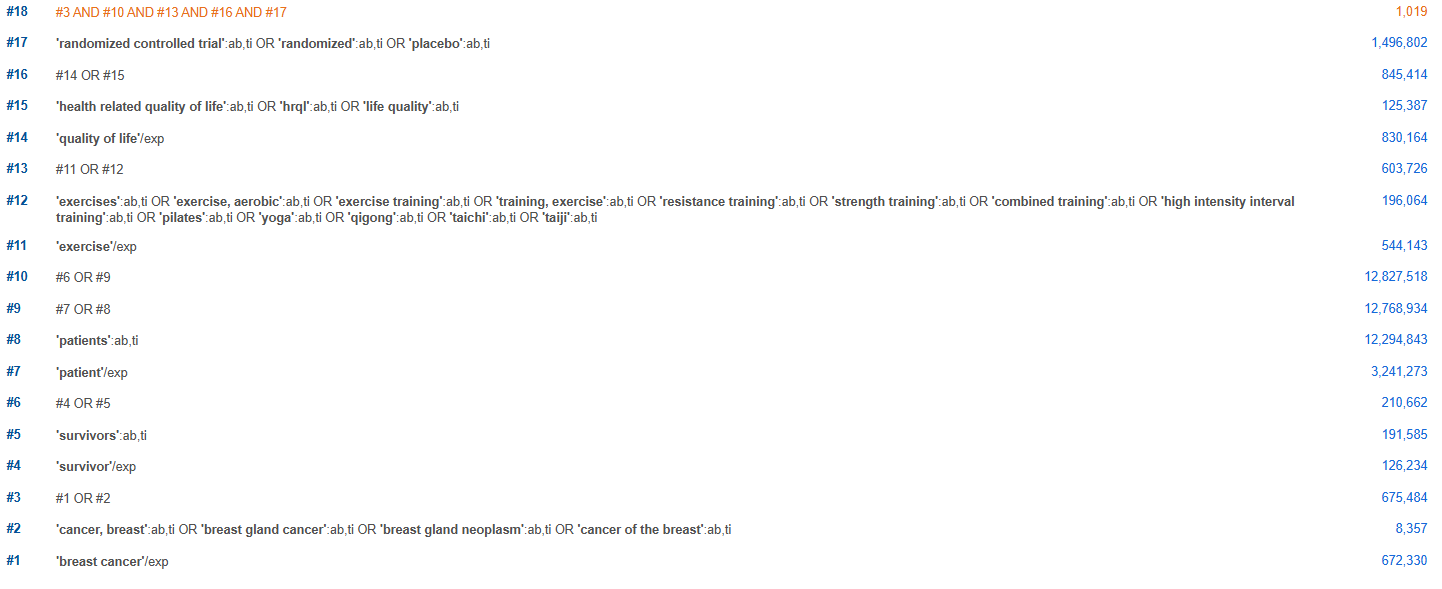
Figure 2 Search strategy in Embase

Table 3 Search strategy in Web Of Science

| Search strategy |
| --- |
| Search:"Breast Neoplasms" or "Breast Neoplasm" or "Breast Cancer" or "Cancer, Breast" or "Cancer of Breast" or "Cancer of the Breast" or "Breast Carcinoma" or "Breast Carcinomas" or "Carcinoma, Breast" or "Carcinomas, Breast" or "Survivors" or "survivor" or "Patients" or "Patient" or "exercise" or "Exercises" or "Exercise, Aerobic" or "Aerobic Exercise" or "Exercises, Aerobic" or "Exercise Training" or "Training, Exercise" or "resistance training " or "strength training" or "combined training" or "high intensity interval training" or "Pilates" or "Yoga" or "qigong" or "taichi " or "taiji" or "Quality of Life" or "Life Quality" or "Health-Related Quality Of Life" or "Health Related Quality Of Life" or "HRQOL" or "randomized controlled trial" or "randomized" or "placebo" |


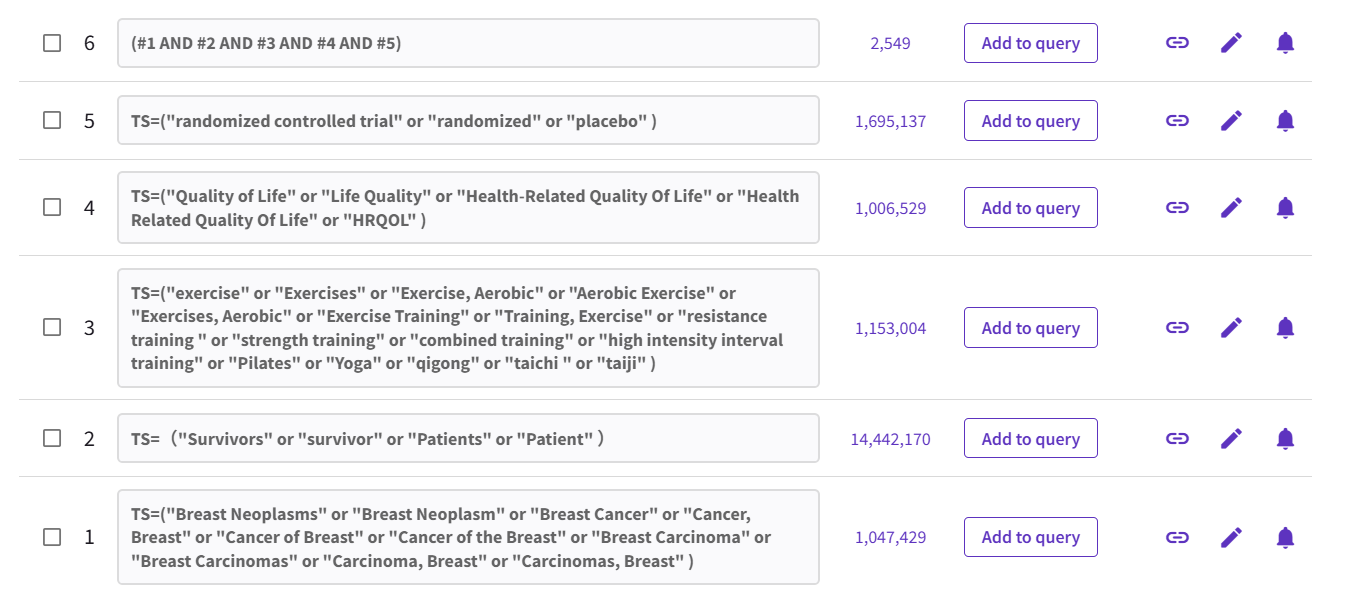


Figure 3 Search strategy in Web Of Science

Table 4 Search strategy in EBSCO

| Search strategy |
| --- |
| Search:(AB Breast Neoplasms OR AB Breast Neoplasm OR AB Breast Cancer OR AB Cancer, Breast OR AB "Cancer of Breast" OR AB "Cancer of the Breast" OR AB Breast Carcinoma OR AB Breast Carcinomas OR AB Carcinoma, Breast OR AB Carcinomas, Breast) AND (AB Survivors OR AB survivor OR AB Patients OR AB Patient) AND (AB exercise OR AB Exercises OR AB Exercise, Aerobic OR AB Aerobic Exercise OR AB Exercises, Aerobic OR AB Exercise Training OR AB Training, Exercise OR AB resistance training OR AB strength training OR AB combined training OR AB high intensity interval training OR AB Pilates OR AB Yoga OR AB qigong OR AB taichi OR AB taiji) AND (AB Quality of Life OR AB Life Quality OR AB Health-Related Quality Of Life OR AB Health Related Quality Of Life OR AB HRQOL) AND (AB randomized controlled trial OR AB randomized OR AB placebo) |


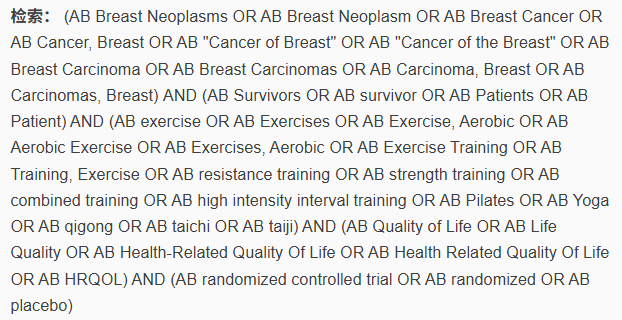


Figure 4 Search strategy in EBSCO

Table 5 Search strategy in Scopus

| Search strategy |
| --- |
| Search:"Breast Neoplasms" or "Breast Neoplasm" or "Breast Cancer" or "Cancer, Breast" or "Cancer of Breast" or "Cancer of the Breast" or "Breast Carcinoma" or "Breast Carcinomas" or "Carcinoma, Breast" or "Carcinomas, Breast" AND "Survivors" or "survivor" or "Patients" or "Patient" or "exercise" AND "Exercises" or "Exercise, Aerobic" or "Aerobic Exercise" or "Exercises, Aerobic" or "Exercise Training" or "Training, Exercise" or "resistance training " or "strength training" or "combined training" or "high intensity interval training" or "Pilates" or "Yoga" or "qigong" or "taichi " or "taiji" AND "Quality of Life" or "Life Quality" or "Health-Related Quality Of Life" or "Health Related Quality Of Life" or "HRQOL" AND "randomized controlled trial" or "randomized" or "placebo" |


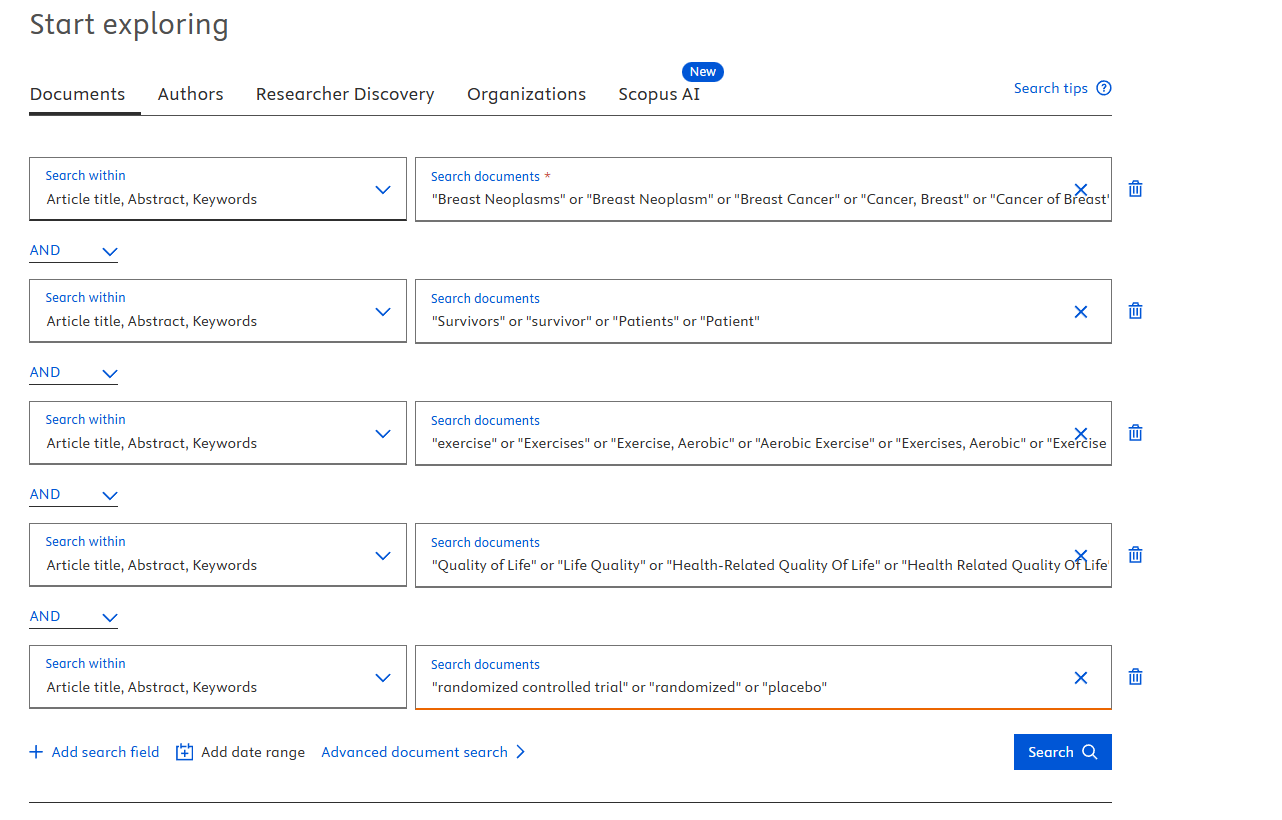
Figure 5 Search strategy in Scopus

Table 6 Search strategy in Cochrane library

| Search strategy |
| --- |
| Search:(Breast Neoplasm):ti,ab,kw or (Breast Cancer):ti,ab,kw or (Cancer, Breast):ti,ab,kw or (Cancer of Breast):ti,ab,kw or (Cancer of the Breast):ti,ab,kw or (Breast Carcinoma):ti,ab,kw or (Breast Carcinomas):ti,ab,kw or (Carcinoma, Breast):ti,ab,kw or (Carcinomas, Breast):ti,ab,kw AND (Survivors):ti,ab,kw or (survivor):ti,ab,kw or (Patients):ti,ab,kw or (Patient):ti,ab,kw AND (Exercises):ti,ab,kw or (Exercise, Aerobic):ti,ab,kw or (Aerobic Exercise):ti,ab,kw or (Exercises, Aerobic):ti,ab,kw or (Exercise Training):ti,ab,kw or (Training, Exercise):ti,ab,kw or (resistance training ):ti,ab,kw or (strength training):ti,ab,kw or (combined training):ti,ab,kw or (high intensity interval training):ti,ab,kw or (Pilates):ti,ab,kw or (Yoga):ti,ab,kw or (qigong):ti,ab,kw or (taichi ):ti,ab,kw or (taiji):ti,ab,kw AND (Life Quality):ti,ab,kw or (Health-Related Quality Of Life):ti,ab,kw or (Health Related Quality Of Life):ti,ab,kw or (HRQOL):ti,ab,kw (randomized controlled trial):ti,ab,kw or (randomized):ti,ab,kw or (placebo):ti,ab,kw |


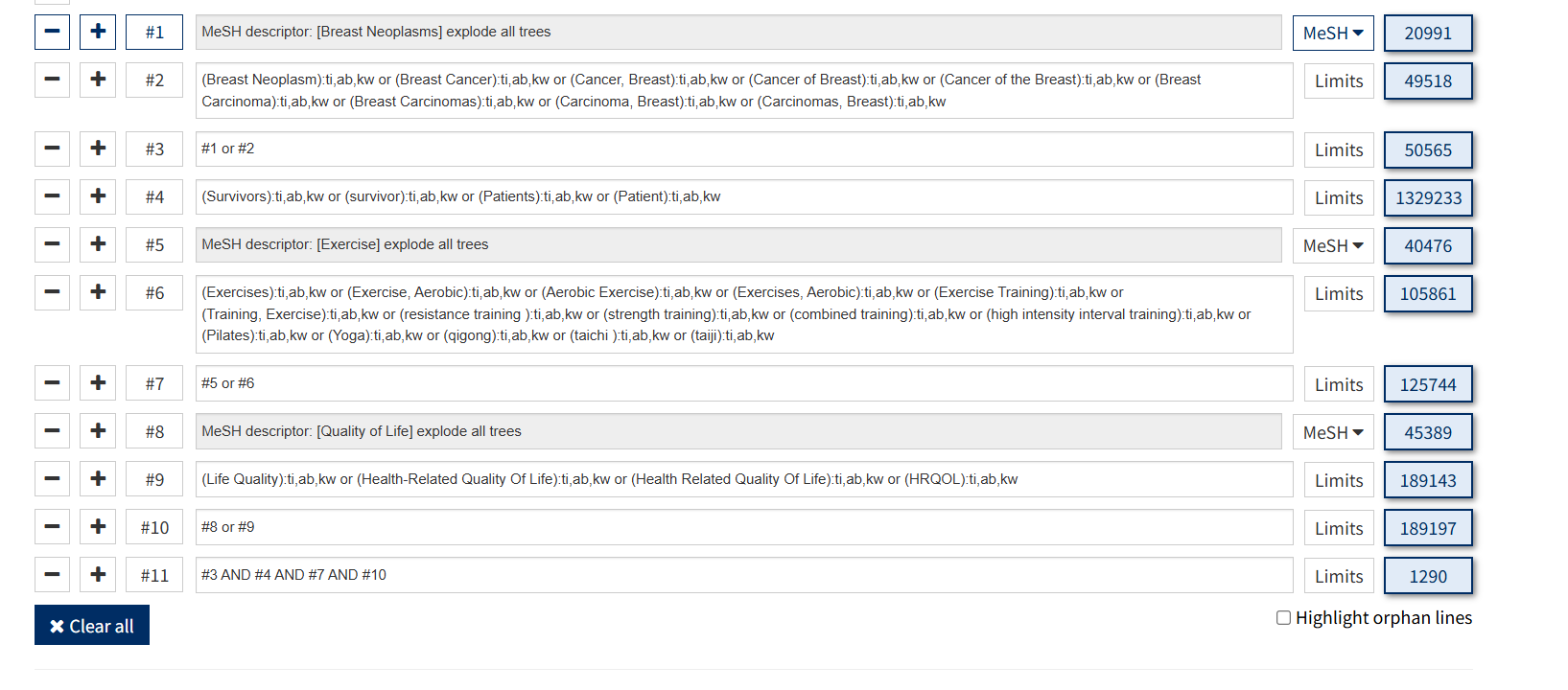
Figure 6 Search strategy in Cochrane library

# Appendix 2 The classifications of exercise interventions

Table 7 classifications of exercise interventions

| Types | Define |
| --- | --- |
| Aerobic exercise | AE, aiming to improve cardiovascular fitness including walking, running, swimming or cycling. |
| Resistance training | RT, with the intent of increasing muscular strength and power using elastic bands, weight-machines or free weights. |
| Combined exercise | CE, the combination of AE and RT. |
| High-Intensity Interval Training | HIIT,A time-efficient training modality characterized by alternating bouts of short-duration high-intensity exercise |
| Yoga | Yoga,A comprehensive exercise program that affects the mind and body, promoting proper body position through concentration, relaxation, respiration, and physical exercise |
| Pilates | Pilates,A exercise modality based on six principles (centering, concentration, precision, breathing techniques and flow during the exercises) |
| Tai Chi | Tai Chi,A mind-body exercise, also known as meditative movement, which incorporates both physical activity and stress reducing aspects |
| Qigong | Qigong,A traditional Chinese mind body exercise incorporating and combining different slow motions and breathing exercises |

# Appendix 3 The measurement tools for Quality Of Life outcomes

Table 8 measurement tools for Quality Of Life

| Outcome |  |
| --- | --- |
| Quality of life | **EORTC QLQ-C30:** European Organization for Research and Treatment of Cancer Core Cancer Quality of Life Questionnaire;  **SF-36:** 36-Item Short Form Health Survey;  **FACT-B:** Functional Assessment of Cancer Therapy-Breast Scale;  **EuroQol-5**:Quality of life was measured by the EuroQol-5 dimensions-5 level questionnaire;  **FACT-G:** Functional Assessment of Cancer Therapy-General;  **FACT-An:** Functional Assessment of Cancer Therapy–Anemia scale;  **CARES-SF:** cancer rehabilitation evaluation system short form. |

# Appendix 4 Study characteristics

| **Study** | **Country** | **Characteristics of subject** | | **Interventions information** | | | | **outcome** | | **condition** |
| --- | --- | --- | --- | --- | --- | --- | --- | --- | --- | --- |
|  |  | **Number (T/C)** | **age (mean[SD])** | **Type of exercise** | **Modality**，**Duration and intensity** | **Period and frequency  (week × [times/week])** | **supervised or nonsupervised** |  | |  |
| Rashid2025 | UAE | 31/31 | 41.10 ± 7.0 | AE+RT(CE) | 60-minute circuit training class (aerobic and resistance combined) | 8×5 | supervised | QLQ-C30 | | Chemotherapy and endocrine treatment |
|  |  |  | 40.54 ± 8.7 | CON | maintained routine life,standard care |  |  |  | |  |
| Bettariga2025 | Australia | 14/14 | 61.8 ± 8.9 | RT | 60–80% 1RM,8–12 repetitions for 3–5 sets of major muscle group | 12×3 | supervised | QLQ-C30 | | Chemotherapy and Radiation therapy |
|  |  |  | 56.9 ± 8.9 | HIIT | performed 5–7 bouts of 30s,60–90% of estimated HRmax |  |  |  | |  |
| Bertoli2025 | Brazil | 21/19 | 55.2 ± 7.6 | Pilates | 60-min sessions, three times weekly | 24×3 | supervised | SF-36,EORT-30 | | hormone therapy |
|  |  |  | 55.4 ± 6.6 | CON | usual care |  |  |  | |  |
| Wong2024 | China | 16/18 | 48.63 ± 8.77 | Yoga | 60-min face-to-face,yogic breathing , postures , and meditation | 8×1 | supervised | FACT-B | | Chemotherapy,Radiotherapy and Hormonal therapy |
|  |  |  | 45.78 ± 9.25 | CON | usual care |  |  |  | |  |
| Min2024 | South Korea | 28/28 | 50.7±6.8 | RT | 20 to 30 minutes,neck stretching, shoulder circle,pendulum exercise, fist clench, rowing, isometric lower limb exercise, and calf raise | 4×4 | supervised | EuroQol-5 | | post-surgery |
|  |  |  | 49.9±6.5 | CON | usual care |  |  |  | |  |
| Li2024 | China | 21/19 | 47.38 ± 1.96 | AE | 50–75% HRmax,treadmill,stationary bike,walking | 12×3 | supervised | FACT-B | | chemotherapy |
|  |  |  | 48.47 ± 2.13 | CON | usual care,education |  |  |  | |  |
| Klavina2024 | Latvia | 17/20 | 48.56 ± 7.84 | HIIT | 4 × 4 minutes at 85%-95% peak heart rate,2 to 3 HIIT sessions per week | 24×3 | supervised | EORTC QLQ-C30 | | chemotherapy |
|  |  |  | 48.53 ± 8.21 | CON | usual care |  |  |  | |  |
| Isanejad2023 | Iran | 10/10/10 | 46.29 ± 6.29 | AE | 50%-60% VO2peak | 12×3 | supervised | FACT-G | | surgery,chemotherapy,radiation therapy |
|  |  |  | 44.00 ± 9.14 | HIIT | 50%-60% VO2peak,90% VO2peak |  |  |  | |  |
|  |  |  | 44.90 ± 5.02 | CON | usual care |  |  |  |  |  |
| Chang2023 | China | 30/30 | 51.91 ± 10.51 | Qigong | Three Postures,35 min per day, 5 days per week, for 15 weeks. | 15×5 | supervised | EORTC QLQ-C30 | | surgery |
|  |  |  | 52.77 ± 8.53 | CON | usual care |  |  |  | |  |
| Yao2022 | Australia | 36/36 | 45.3 ± 8.5 | Tai chi | two 60 min sessions per week，8-form Yang style Tai chi | 8×2 | supervised | FACT-B | | chemotherapy |
|  |  |  | 48.6 ± 7.8 | CON | usual care |  |  |  | |  |
| Wei2022 | China | 35/35 | 52(43, 60) | Qigong(Baduanjin) | stretching the joints, inhalation and exhalation for 2 min each, and two 12-min Baduanjin sessions | 12×5 | supervised | FACT-B | | surgery |
|  |  |  | 55 (50, 62) | CON | usual care |  |  |  | |  |
| Liu2022 | China | 68/68 | 18-60 | YOGA | 90 min of weekly,mindfulness meditation,yoga postures,body scan,sensory summary | 8×1 | supervised | FACT-B | | surgery |
|  |  |  | 18-60 | CON | usual care |  |  |  | |  |
| Liao2022 | China | 33/35 | 53.12 ± 7.02 | Qigong(Baduanjin) | 8 postures,90 min per session with 2 sessions per week | 12×2 | supervised | EORTC QLQ-C30 | | Aromatase inhibitors treatment |
|  |  |  | 54.63 ± 8.44 | CON | usual care |  |  |  | |  |
| Moraes2021 | Brazil | 12/13 | 55.0 ± 5.8 | RT | 45° leg press, stiff-legged dead lifts, bench press, lat pulldown, and sit-ups | 8×1 | supervised | SF-36 | | surgery, radiotherapy, and chemotherapy |
|  |  |  | 54.3 ± 5.2 | CON | remained physically inactive |  |  |  | |  |
| Eyigör2021 | Turkey | 15/16 | 51.40 ± 10.6 | YOGA | 5 min for meditation,10 min for Shavasana,Seven postures | 10×2 | supervised | EORTC QLQ-C30 | | posttreatment |
|  |  |  | 50.7±7.6 | CON | usual care |  |  |  | |  |
| Aydin2021 | Turkey | 24/24 | 40-60 | AE+RT(CE) | 40-minute leg and hip workout by using elastic band and ball;50-60%HR,walking and cycling |  | supervised | EORTC-QLQ-C30 | | surgery |
|  |  |  |  | CON | usual care |  |  |  | |  |
| Ying2019 | China | 46/40 | 36-72 | Qigong(Baduanjin) | 8 postures,session lasted around 60 min | 24×3 | supervised | FACT-B,FACT-G | | surgery, chemotherapy, and/or radiation |
|  |  |  |  | CON | daily physical activity |  |  |  | |  |
| Eyigor2018 | Turkey | 22/14 | 52.3 ± 9.5 | Yoga | 2 days a week, each lasting 1 h,for a period of 10 weeks,nine postures | 10×2 | supervised | EORTC QLQ-C30 | | surgical treatment, radiotherapy, and/or chemotherapy |
|  |  |  | 51.5 ± 7.3 | CON | usual care |  |  |  | |  |
| Dieli2018 | USA | 46/45 | 53.5 ± 10.4 | AE+RT(CE) | 150 min of aerobic exercise and 2–3 days of resistance exercise training/week | 16×3 | supervised | FACT-B | | postsurgery radiotherapy and chemotherapy |
|  |  |  |  | CON | usual care |  |  |  | |  |
| Shobeiri2016 | Iran | 30/30 | 42.7±9.6 | AE | walking, stretching physical activity, and specific movements of arms and shoulders; 50%–75%HRR. | 10×2 | supervised | EORTC QLQ-C30 |  | completed surgery and chemotherapy or radiotherapy |
|  |  |  | 43.5±8.6 | CON | not advised to change habitual activity levels,usual medical care |  |  |  | |  |
| Ligibel2016 | USA | 47/51 | 49.3 ± 9.6 | AE | 150 minutes of moderateintensity exercise per week | 16 | supervised | EORTC QLQ-C30 | | not amenable to surgical resection |
|  |  |  | 50.7 ± 9.4 | CON | usual care |  |  |  | |  |
| Hagstrom2016 | Australia | 19/15 | 51.2 ± 8.5 | RT | three times per week,60 min per session.included leg extension, leg curl or Romanian deadlift, lat pull down, machine bench press, seated row, back extension, prone hold or sit ups. | 16×3 | supervised | FACT-G | | completed surgery, radiotherapy and/or chemotherapy |
|  |  |  | 52.7 ± 9.4 | CON | not advised to change habitual activity levels,usual medical care |  |  |  | |  |
| Galiano2016 | Spain | 40/41 | 47.4±9.6 | AE+RT(CE) | warm-up,resistance and aerobic exercise training, and 3) cooldown | 24×3 | supervised | EORTC QLQ-C30 |  | completed adjuvant therapy |
|  |  |  | 49.2±7.9 | CON | Usual care |  |  |  | |  |
| De Luca2016 | Italy | 10/10 | 50.2 ± 9.7 | AE+RT(CE) | two weekly sessions of 90-min;RT:40%-60% of 1RM;AE:stationary bike,20 min at 70% of the estimated HRmax | 24×2 | supervised | FACT-G | | postsurgery radiotherapy and chemotherapy |
|  |  |  | 46 ± 2.8 | CON | usual lifestyle |  |  |  | |  |
| Adams2016 | Canada | 109/91/51 | 48.8(25–78) | AE | 60 mins(15 mins at 60% of VO2peak,and 45 mins at 80 % of VO2peak),treadmill, cycle ergometer, or elliptical-based exercise. | 17×3 | supervised | FACT-An | | Chemotherapy treatment |
|  |  |  |  | RT | 2 sets of 8–12 repetitions of 9 exercises,60 and 70 % of their predicted 1RM |  |  |  | |  |
|  |  |  |  | CON | avoid any new exercise training |  |  |  | |  |
| Stan2016 | USA | 18/16 | 61.4 ± 7.0 | YOGA | relaxed breathing, soft yoga, standing yoga, chair stretches, and guided | 12×3 | supervised | FACT-B | | Chemotherapy,endocrine treatment,Radiation,Mastectomy |
|  |  |  | 63.0 ± 9.3 | RT | upper and lower extremities as well as core muscles with resistance provided by elastic bands. |  |  |  | |  |
| Travier2015 | Netherlands | 87/77 | 49.7 ± 8.2 | AE+RT(CE) | AE:HR at (3×2 min increasing to 2×7 min) or below (3×4 min decreasing to 1×7 min) ventilatory threshold.RT:arms, legs, shoulder, and trunk;1×20 repetitions(45%1RM)-2×10 repetitions(65%1RM)-1×10 repetitions(75%1RM) | 18×2 | supervised | EORTC QLQ-C30 | | received chemotherapy |
|  |  |  | 49.5 ± 7.9 | CON | usual care |  |  |  | |  |
| Schmidt2015 | Germany | 49/46 | 52.2 ± 9.9 | RT | 8 different machine-based progressive resistance exercises,8–12 repetitions at 60–80% of 1RM | 12×2 | supervised | EORTC QLQ-C30 | | adjuvant chemotherapy |
|  |  |  | 53.3 ± 10.2 | CON | loosening all muscles |  |  |  | |  |
| Carmer2015 | Germany | 19/21 | 48.3±4.8 | YOGA | 90-minute,yoga postures (including child pose, cobra pose, corpse pose, crocodile pose, fish pose, forward bend, half bridge pose, half twist pose, shoulder stand, staff pose, sun salutation) | 12×1 | supervised | FACT-B | | had completed surgical |
|  |  |  | 50.0±6.7 | CON | usual care |  |  |  | |  |
| Casla2015 | Spain | 47/47 | 45.91 ± 8.21 | AE+RT(CE) | AE:55%-85%HRR,walking and running.RT:Shoulder circles, dorsal and chest exercises with elastic bands;10-20repetitions | 12×2 | supervised | SF-36 | | completion of radiotherapy and chemotherapy |
|  |  |  | 51.87 ± 8.21 | CON | usual care |  |  |  | |  |
| Murtezani2014 | Serbia | 30/32 | 53 ± 11 | AE | 50‐75% HRR,three exercise modalities (treadmills, stationary bicycles, and stair‐climbing machines) | 10×3 | supervised | FACT-B | | surgery, radiotherapy, and/or chemotherapy |
|  |  |  | 51 ± 11 | CON | maintain their lifestyle |  |  |  | |  |
| Loh2014 | Malaysia | 32/31/32 | 18–65 | QIGONG | A low-moderate intensity internal Qigong programme, | 8×1 | supervised | FACT-B | | completed primary cancer treatmen |
|  |  |  |  | AE | four sets of aerobic movements that were taught face-to-face once a week (90-minute sessions for 8 weeks) |  |  |  | |  |
|  |  |  |  | CON | usual care |  |  |  | |  |
| Ergun2013 | USA | 20/20/20 | 55.05±6.85 | AE | brisk walking for 30 min/day | 12×3 | supervised | EORTC QLQ-C30 |  | completion of surgical, radiation therapy and chemotherapy |
|  |  |  | 50.3±10.37 | AE+RT(CE) | doctor supervised exercise group and brisk walking |  |  |  | |  |
|  |  |  | 49.65±8.25 | CON | usual care |  |  |  | |  |
| Chen2013 | China | 49/47 | 45.3±6.3 | QIGONG | walking qigong,Preparation,Main and Ending Exercise | 12×5 | supervised | FACT-G | | had undergone surgery |
|  |  |  | 44.7±9.7 | CON | usual care |  |  |  | |  |
| Campo2013 | USA | 29/25 | 66.54 (55–89) | Tai chi | consisted of 19 simple, repetitive, non-strenuous movements and one standing pose | 12×3 | supervised | SF-36 | | Chemotherapy was completed |
|  |  |  | 65.64(57–84) | CON | usual care |  |  |  | |  |
| Sprod2012 | USA | 9/10 | 54.33±3.55 | Tai chi | 40 min of Yang-style tai chi chuan using the 15-move | 12×3 | supervised | MOS SF-36 | | treatment completed |
|  |  |  | 52.70±2.11 | CON | standard support Therapy |  |  |  | |  |
| Littman2012 | USA | 32/31 | 60.6 ± 7.1 | Yoga | including centering exercises, seated and standing poses, relaxation, breathing exercises, and meditation. | 24×5 | supervised | FACT-G | | postsurgery radiotherapy and chemotherapy |
|  |  |  | 58.2 ± 8.8 | CON | Usual care (waitlist) |  |  |  | |  |
| Eyigor2010 | Turkey | 27/15 | 48.52±7.62 | pilates | pilates exercises including one leg stretch, double leg stretch, shoulder bridge, arm opening, hundreds, clam, hip twist and side kick (2 sets, 10 repetitions) | 8×3 | supervised | EORTC QLQ-C30 | | completion of treatment with surgery, radiotherapy and/or chemotherapy |
|  |  |  | 49.73±8.71 | CON | usual care |  |  |  | |  |
| Rogers2009 | Canada | 21/20 | 52 ± 15 (36–68) | AE | walking, moderate intensity. | 12×3-5 | supervised | FACT-B | | remain on hormonal therapy |
|  |  |  | 54 ± 8 (36–68) | CON | usual care |  |  |  | |  |
| Milne2008 | Australia | 29/29 | 55.2±8.4 | AE+RT(CE) | AE:cycle and rowing ergometers,the mini-trampoline, and the step-up blocks.RT:chest press, chest extension, biceps curls, triceps extension, leg extension, leg curls, hip abduction and adduction, back extension,abdominal crunches, standing fly’s and leg press. | 12×3 | supervised | FACT-B | | completing adjuvant therapy |
|  |  |  | 55.1±8.0 | CON | usual care |  |  |  | |  |
| Moadel2007 | USA | 84/44 | 55.11 ± 9.95 | Yoga | Hatha yoga techniques,physical stretches and poses; breathing exercises; and meditation. | 12×12 | supervised | FACT-G | | Chemotherapy treatment |
|  |  |  | 54.23 ± 10.07 | CON | usual care |  |  |  | |  |
| Courneya2007 | Canada | 78/82/82 | 49(25–78) | AE | three times per week on a cycle ergometer, treadmill, or elliptical | 17 | supervised | FACT-An | | beginning first-line adjuvant chemotherapy |
|  |  |  |  | RT | three times per week performing two sets of eight to 12 repetitions of nine different exercises at 60% to 70% of their estimated onerepetition maximum. |  |  |  | |  |
|  |  |  |  | CON | standard care |  |  |  | |  |
| Culos2006 | Canada | 18/18 | ＞18 | Yoga | a series of 6–10 modified Yoga asanas | 7 | supervised | EORTC QLQ-C30 | | not currently undergoing active treatment |
|  |  |  |  | CON | usual care |  |  |  | |  |
| Courneya2003 | Canada | 24/28 | 59±5 | AE | trained on cycle ergometers | 15×3 | supervised | FACT-B | | had completed surgery, radiotherapy |
|  |  |  | 58±5 | CON | usual care |  |  |  | |  |
|  |  |  |  |  |  |  |  |  | |  |
| Swisher2015 | USA | 13/10 | 53.8 ± 10 | AE | moderate-intensity,60–75 % of peak heart rate | 12×3 | supervised | FACT-B | | After active treatment |
|  |  |  | 53.6 ± 12 | CON | usual medical care |  |  |  | |  |
| Lahart2016 | Uk | 37/33 | 52.4 ± 10.3 | AE | moderate-intensity physical activity | 24×3 | supervised | FACT-B | | post-surgery |
|  |  |  | 54.7 ± 8.3 | CON | usual care |  |  |  | |  |
| Courneya2011 | Canada | 160/160 | 50–70 | AE | moderate-to-vigorous intensity aerobic exercise | 12×5 | supervised | SF-36 | | postoperative |
|  |  |  |  | CON | usual care |  |  |  | |  |
| Adams-C2023 | USA | 15/15 | 63.3 ± 3.2 | AE | moderate-intensity physical activity | 8×5 | supervised | FACT-B | | Radiotherapy treatment |
|  |  |  | 64.5 ± 3.2 | CON | maintain their current daily activities and exercise habits |  |  |  | |  |
| Steindorf2014 | Germany | 77/78 | 55.2 ± 9.5 | RT | eight different machine-based resistance exercises | 12×2 | supervised | EORTC QLQ-C30 | | Chemotherapy treatment |
|  |  |  | 56.4 ± 8.7 | CON | muscle relaxation |  |  |  | |  |
| Soriano2023 | Spain | 32/28 | 52.6 ± 8.8 | RT | 4 dynamic resistance exercises | 12×2 | supervised | FACT-B | | After treatment with chemotherapy or radiotherapy |
|  |  |  | 52 ± 9.4 | CON | undertake ≥10,000 steps |  |  |  | |  |
| Ceˇseiko2019 | Latvia | 27/28 | 48.2 ± 6.7 | RT | 2 warm-up sets followed by RT in 4 sets with 4 repetitions | 12×2 | supervised | EORTC QLQ-C30 | | completed surgery, radiotherapy and/or chemotherapy |
|  |  |  | 49 ± 8 | CON | standard care |  |  |  | |  |
| Paulo2019 | Brazil | 18/18 | 63.2 ± 7.1 | AE+RT(CE) | 40 min of RTon machines followed by 30 min of AE on a treadmill | 36×3 | supervised | FACT-B | | aromatase inhibitor therapyr |
|  |  |  | 66.6 ± 9.6 | CON | relaxation exercises |  |  |  | |  |
| Herrero2006 | Spain | 10/10 | 54.4 ± 10.3 | AE+RT(CE) | RT included 11 exercises,AE for 20 min at 70% of the HRmax | 8×3 | supervised | EORTC QLQ-C30 | | After radiation therapy or after chemotherapy |
|  |  |  | 52.6 ± 10.2 | CON | usual care |  |  |  | |  |
| Pasyar 019 | Iran | 12/15 | 51.6 ± 10.46 | YOGA | 20 asana yoga exercises and 5 breathing exercises | 8×3 | supervised | EORTC QLQ-C30 | | Chemotherapy treatment |
|  |  |  | 51.8 ± 11.4 | CON | routine care |  |  |  | |  |
| Leite2024 | USA | 18/16 | 55.29(10.93 ± 11) | Pilates | general warm-up,main part and cool-down | 16×3 | supervised | EORTC QLQ-C30 | | Participated in any chemotherapy or radiation |
|  |  |  |  | CON | routine care |  |  |  | |  |
|  |  |  |  |  |  |  |  |  | |  |
| Mustian2008 | USA | 11/10 | 52±9 | Tai chi | 40 min and 15-move short-form | 12×3 | supervised | SF-36 | | had received surgical treatment |
|  |  |  |  | CON | Psychosocial support therapy |  |  |  | |  |
| Loudon2014 | Australia | 15/13 | 55.1 ± 2.5 | YOGA | breathing practices, physical postures, meditation and relaxation techniques | 8×7 | supervised | LYMQOL | | had completed treatment |
|  |  |  | 60.5± 3.6 | CON | Usual self-care |  |  |  | |  |
| Kiecolt2014 | USA | 100/100 | 51.8±9.8 | YOGA | on the floor (10 poses), standing (6 poses), seated (in chair or on blankets on floor, 3poses), restorative (4 poses) and breathing practices. | 12×2 | supervised | SF-36 | | had completed cancer treatment |
|  |  |  | 51.3±8.7 | CON | Usual care (waitlist) |  |  |  | |  |
| Speck2010 | USA | 113/120 | 55.5±8 | RT | core exercises to strengthen abdominal and back muscles and weight-lifting exercises; 3*10 rep/exercise | 48×2 | supervised | SF-36 | | Chemotherapy or Radiation |
|  |  |  | 57.5±8.5 | CON | Usual care (waitlist) |  |  |  | |  |
| Ohira2006 | USA | 43/43 | 53.3 ±8.7 | RT | Nine common weight-training exercises were performed using variable resistance machines | 24×2 | supervised | CARES-SF | | Chemotherapy or Radiation |
|  |  |  | 52.8 ±7.6 | CON | Usual care |  |  |  | |  |
| DO2015 | Korea | 32/30 | 47.1±8.5 | AE+RT(CE) | AE:treadmill, bicycle, and stepper machine , 40%–75% VO2max; RT:elbow flexor, hip flexor, hip abductor, hip extensor,knee extensor, knee flexor, 2*8–12 rep/exercise at 60%–80% of 1 RM. | 4×5 | supervised | EORTC QLQ-C30 | | underwent radiation therapy |
|  |  |  | 48.3±8.2 | CON | Usual care (waitlist) |  |  |  | |  |
| Cerulli2014 | Italy | 10/10 | 45.3±4.32 | AE | riding | 16×2 | supervised | FACIT-F | | all cancer-related treatments |
|  |  |  | 46.0±2.78 | CON | Usual care |  |  |  | |  |
| Rogers2015 | UK | 110/112 | 54.9 ± 9.3 | AE | walking on the treadmill, 40%–59%HRR | 12×3-4 | supervised | FACT-B | | post-surgical |
|  |  |  | 53.9 ± 7.7 | CON | usual care |  |  |  | |  |
| Pinto2015 | USA | 39/37 | 55.64±8.59 | AE | brisk walking, moderate-intensity | 12×4 | supervised | FACT-B | | completed surgery |
|  |  |  | 55.59±10.59 | CON | Information and support |  |  |  | |  |
| Odynets2019 | Ukraine | 45/40/30 | 58.84 ± 1.36 | AE | water exercise, 45%–60% HRR | 48×3 | supervised | FACT-B+4 | | post-surgery |
|  |  |  | 59.40 ± 1.24 | pilates | floor and included warmup, a main part using a resistance band, and cool-down. |  |  |  | |  |
|  |  |  | 59.10 ± 1.37 | Yoga | asana techniques, breathing exercises, and after successful assimilation |  |  |  | |  |
| Baglia2020 | USA | 61/60 | 62.0±7.0 | AE+RT(CE) | gym-based AE and RT. | 48×3-5 | supervised | FACT-B | | chemotherapy and/or radiotherapy |
|  |  |  | 60.5±7.0 | CON | Usual care |  |  |  | |  |
| Uth2020 | Denmark | 46/22 | 47.4±9.4 | AE | Each session consists of a warm-up (e.g., running, squats, sit-ups, back extensions,and balance exercises), football drills (passing, dribbling, and shooting) and 3–4*7 min of small-sided games. | 48×2 | supervised | SF-36 | | completion adjuvant chemotherapy and/or radiation |
|  |  |  | 50.0±9.3 | CON | Usual care |  |  |  | |  |
| Buchan2016 | USA | 21/20 | 53.7±10.54 | AE | walking/jogging,cycling, swimming; at a MET level of 3–3.5, increasing to 5. | 12 | supervised | FACT-B | | had completed treatment |
|  |  |  | 58.5±9.19 | RT | fullbody strength training program, including chest fly, triceps kick-back, squat, curl-ups, bent-over row, bridging, wall push-up, bicep curls, calf raises, shoulder press, external rotation and forward lunge, 2*8–12rep/exercise, MET level of 3–3.5 to 5 |  |  |  | |  |

Table 9 Basic characteristics of the included literature.

# Appendix 5 The Classification of exercise parameters for subgroup analyses

Table 10 Classification of exercise parameters.

| Frequency | |
| --- | --- |
| Low | <3 times/week |
| High | ≥3 times/week |
| Duration per session |  |
| Short | ≤40 minutes |
| long | ＞40 minutes |
| Length of intervention |  |
| Short | ≤8 weeks |
| long | ＞8 weeks |
| Intensity | |
| Low | Aerobic exercise:  < 60%HR max/60% V̇O_2_max  Resistance exercise: < 50% of 1 RM |
| Moderate | Aerobic exercise: 60%-85% HR max/60–80% V̇O_2_max  Resistance exercise: 60–80% of 1 RM |
| High | Aerobic exercise: >85% HR max/80% V̇O_2_max,  Resistance exercise: 80–100% of 1 RM |

| Frequency | |
| --- | --- |
| Low | 1-2 times/week |
| Moderate | 3-4 times/week |
| High | ≥5 times/week |
| Duration per session |  |
| Short | <30 minutes |
| Moderate | 30-59 minutes |
| long | ≥60 minutes |
| Length of intervention |  |
| Short | <12 weeks (3 months) |
| Moderate | 13-24 weeks (3 months-6 months) |
| long | ≥25 weeks (6 months) |
| Intensity | |
| Low | Aerobic exercise:  < 60%HR max/60% V̇O_2_max  Resistance exercise: < 50% of 1 RM |
| Moderate | Aerobic exercise: 60%-85% HR max/60–80% V̇O_2_max  Resistance exercise: 60–80% of 1 RM |
| High | Aerobic exercise: >85% HR max/80% V̇O_2_max,  Resistance exercise: 80–100% of 1 RM |

#
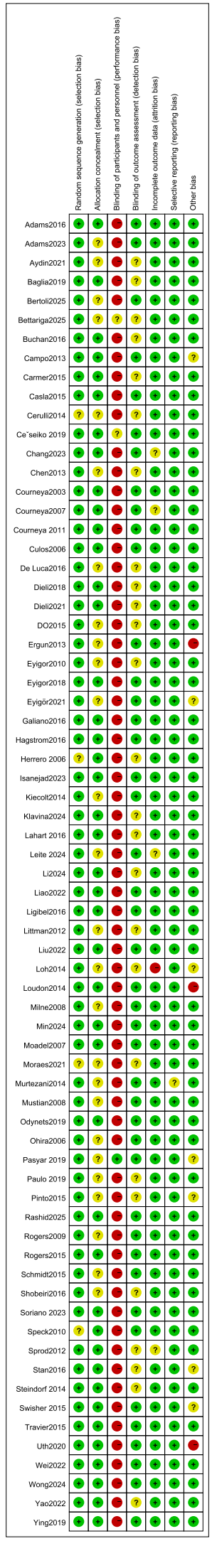
Appendix 6 Risk of bias Summary

Figure 7 Risk of bias summary

# Appendix 7 The analysis for outcome

## 7.1.1 Pairwise analysis for Quality Of Life outcome


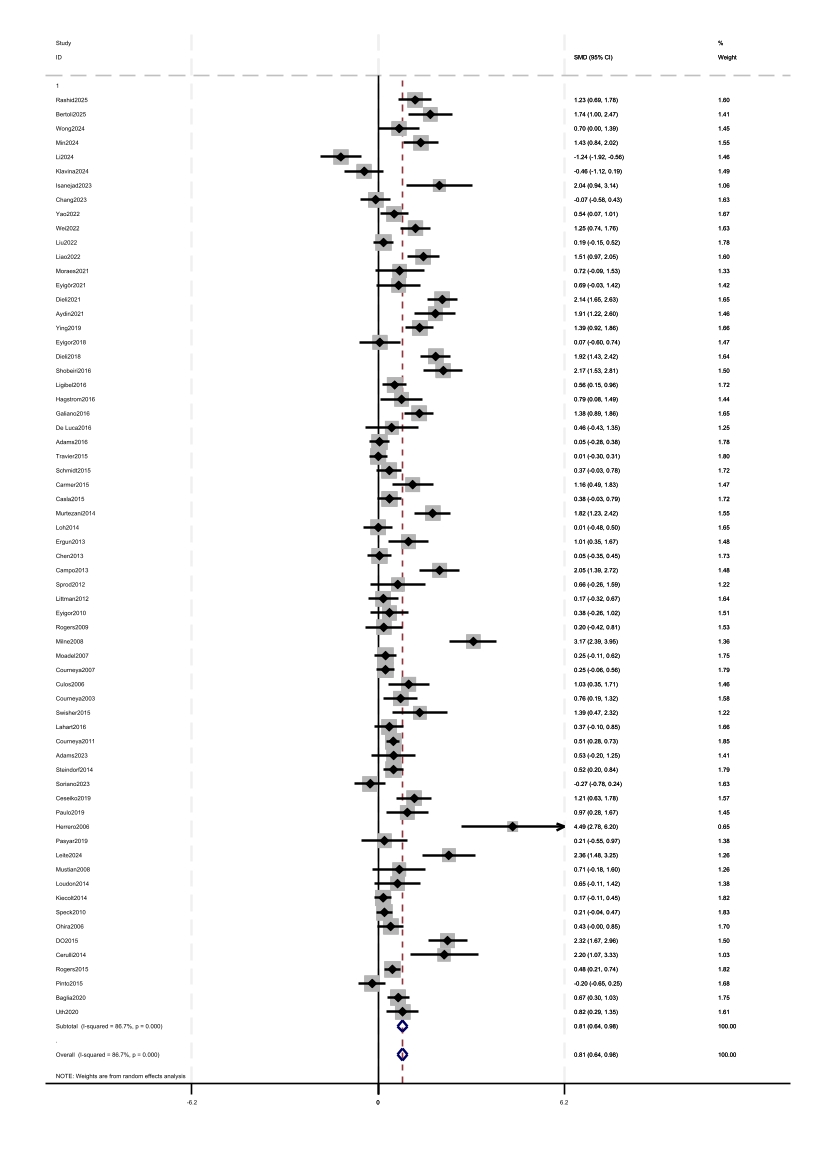


Figure 8 Forest plot of pairwise meta-analysis for quality of life

## 7.1.2 Pairwise analysis of quality of life results for different exercises


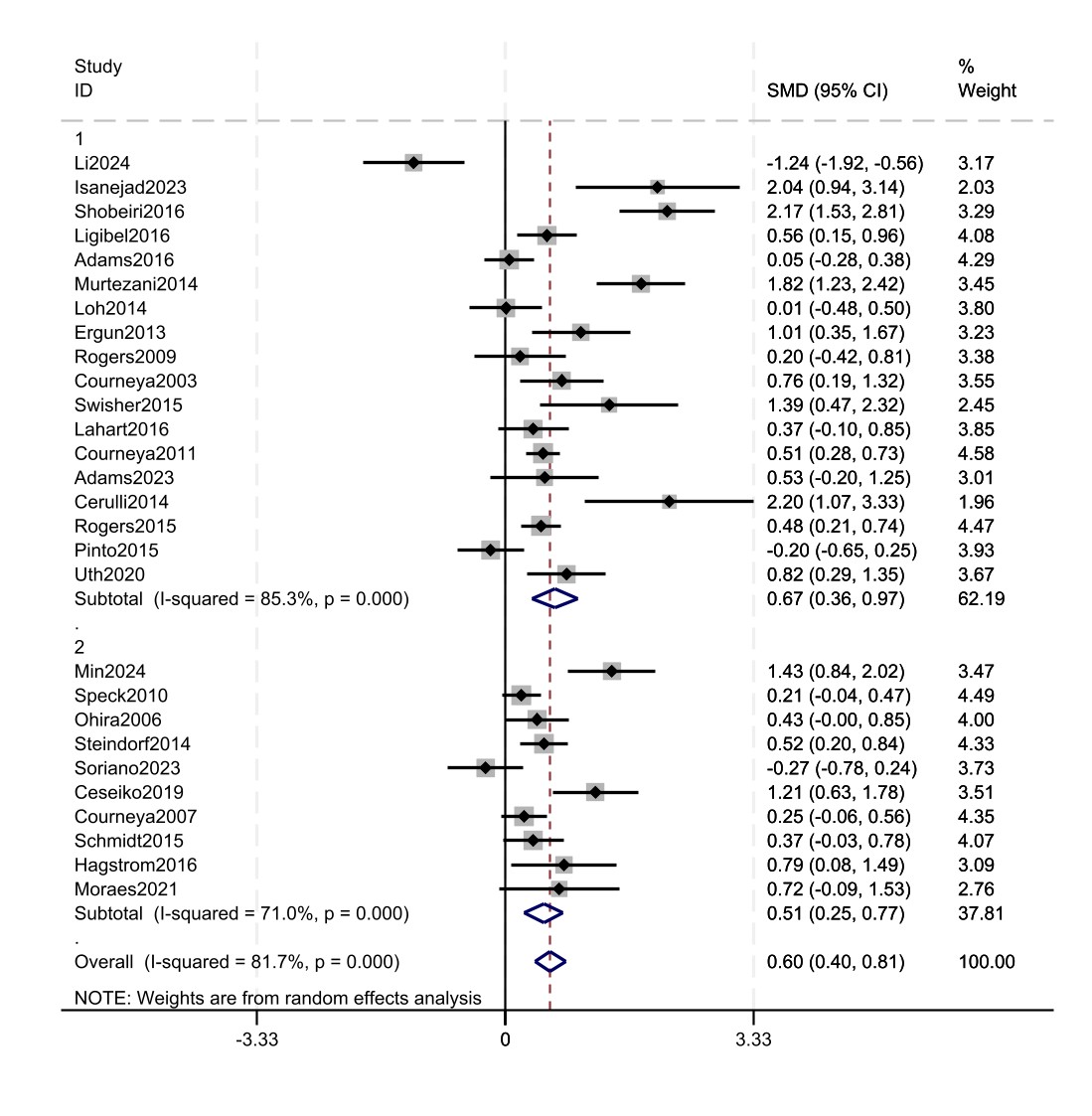


Note:1:AE,2:RT


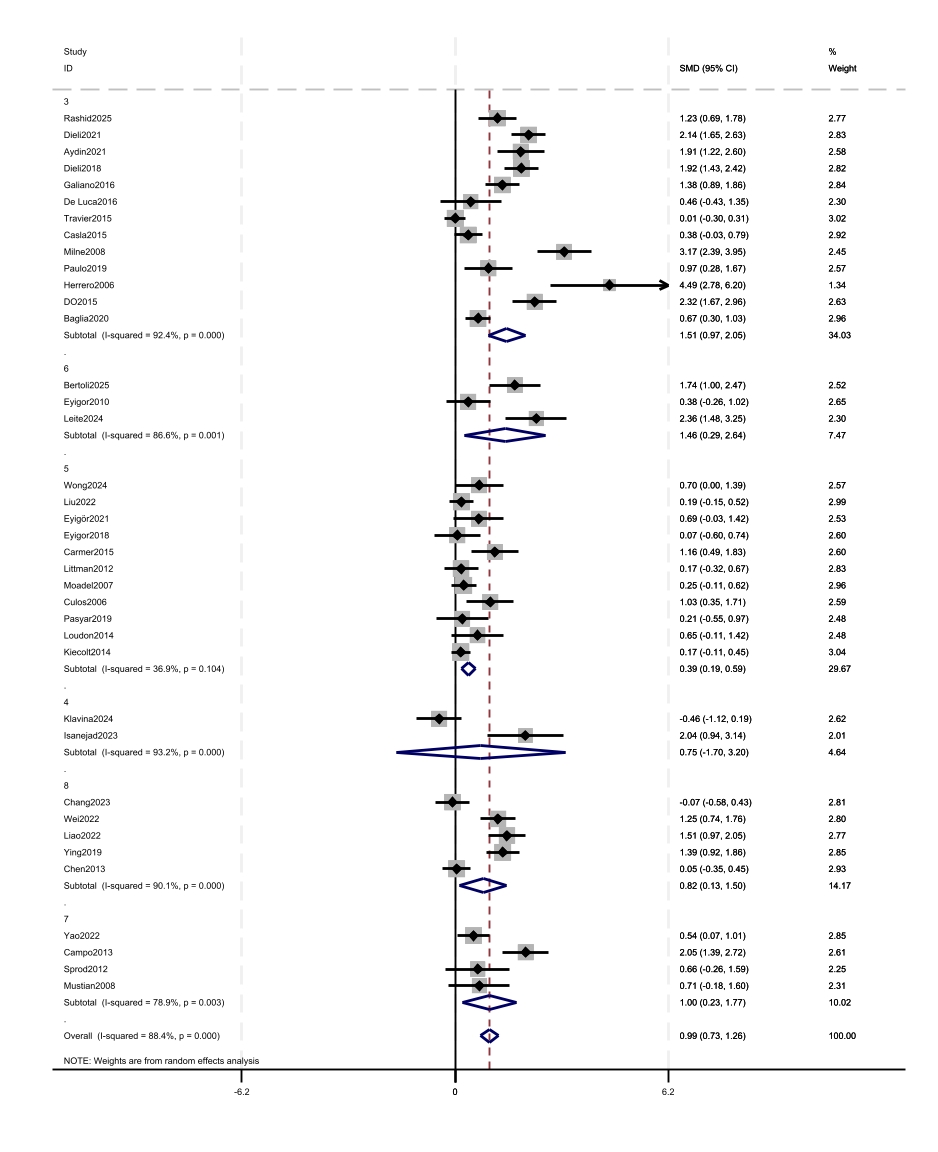


NOTE:3:CE;4:HIIT;5:YOGA;6:PILATES;7:TAICHI;8:QIGONG

## 7.2 Inconsistency test for quality of life

**Loop inconsistency test in quality of life：**


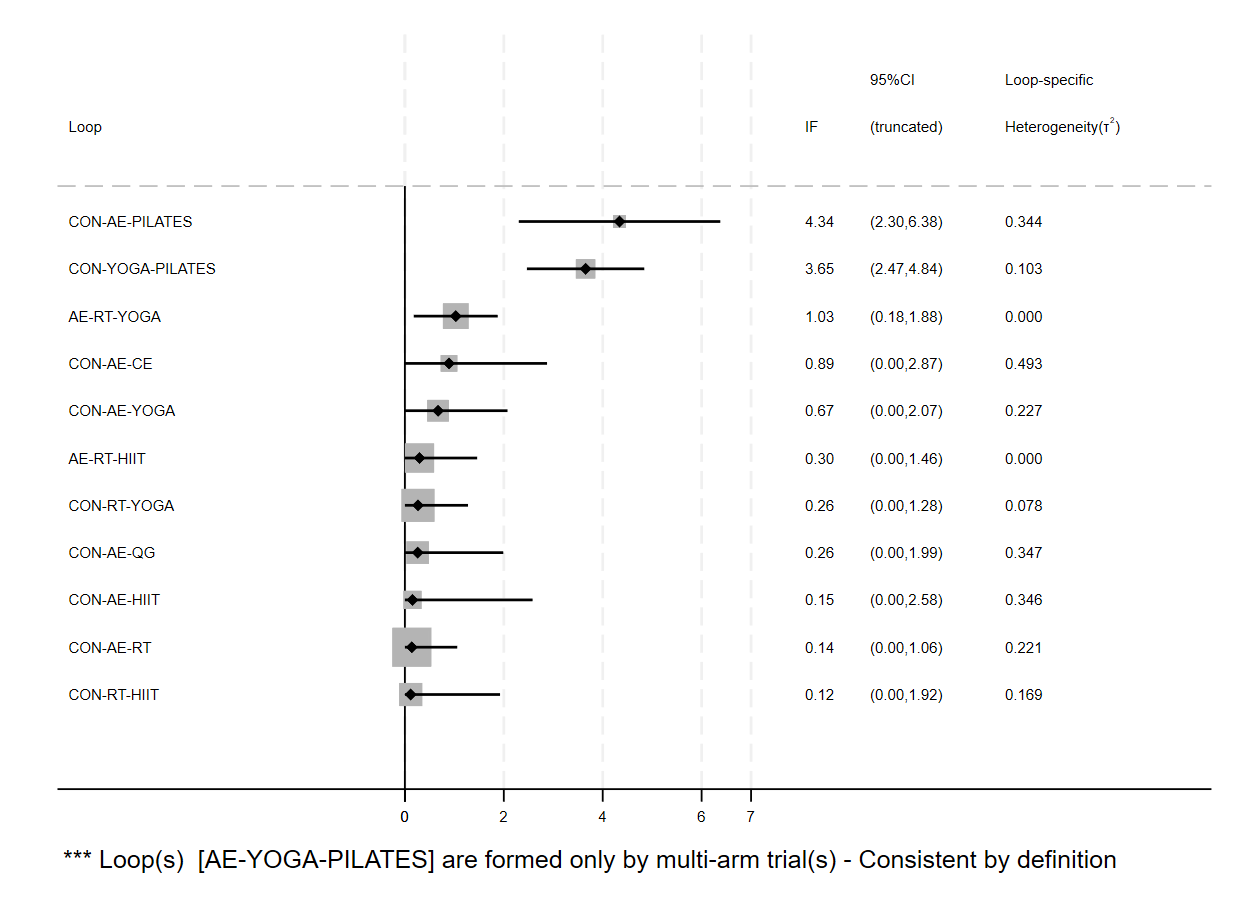


Figure 8 Loop inconsistency test

## 7.3 Predictive interval plot for the quality of life network


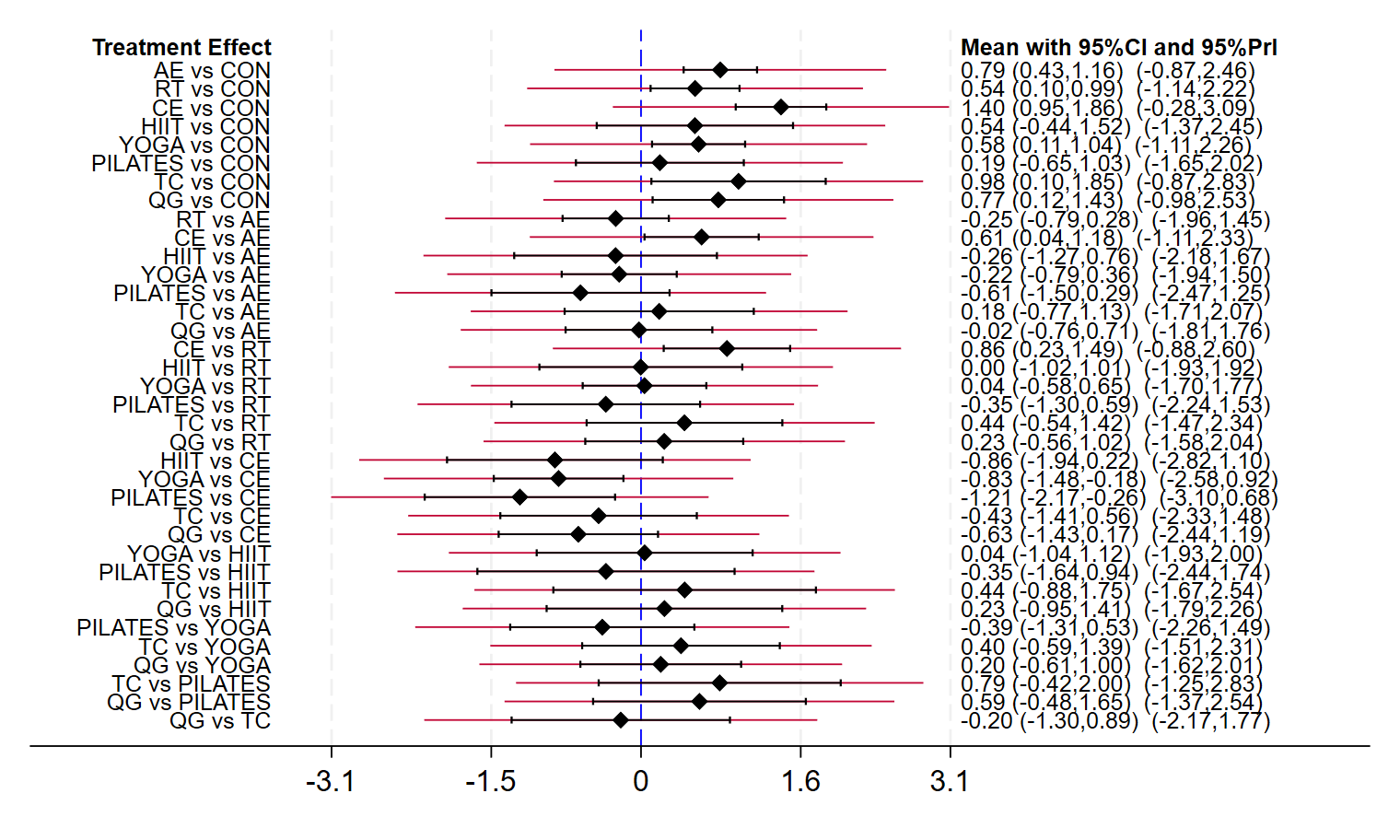
Figure 9 Predictive interval plot for the quality of life network. CON: control; AE: aerobic exercise; RT: resistance training; CE: combined exercise;TC: Taichi; QG: Qigong

## 7.4 Treatment Relative Ranking of Model


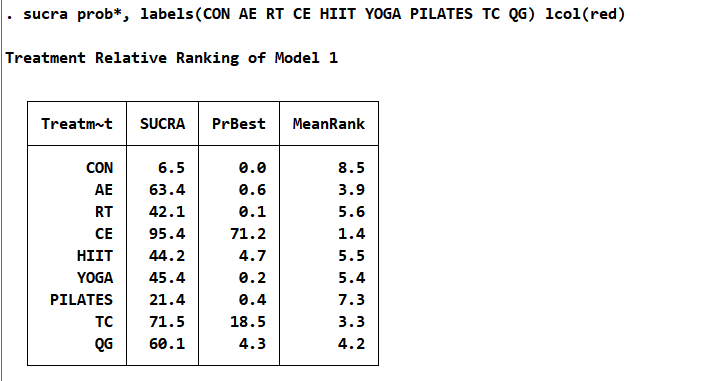


Figure 10 Treatment Relative Ranking

CON: control; AE: aerobic exercise; RT: resistance training; CE: combined exercise;TC: Taichi; QG: Qigong

# Appendix 8 The analysis for subgroup analysis

## 8.1 SUCRA curves and league tables under different movement parameters


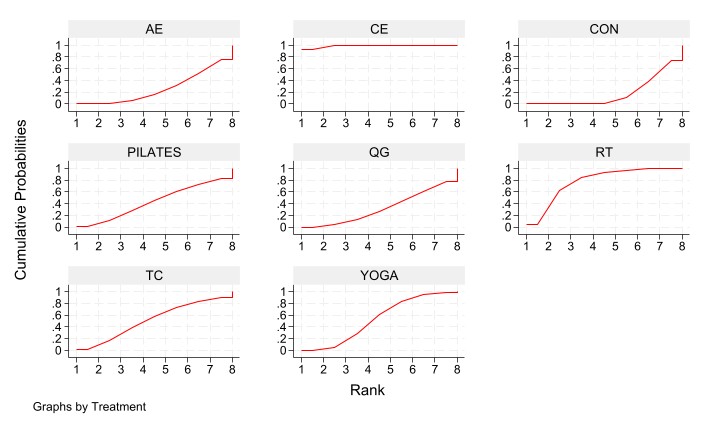


Figure 11 Cumulative ranking probability graph of quality of life after different exercise interventions less than eight weeks.

Figure 1
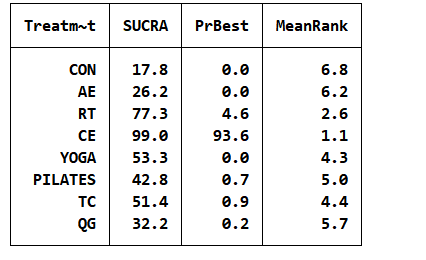
2 Ranking of quality of life for different types of exercise less than eight weeks.


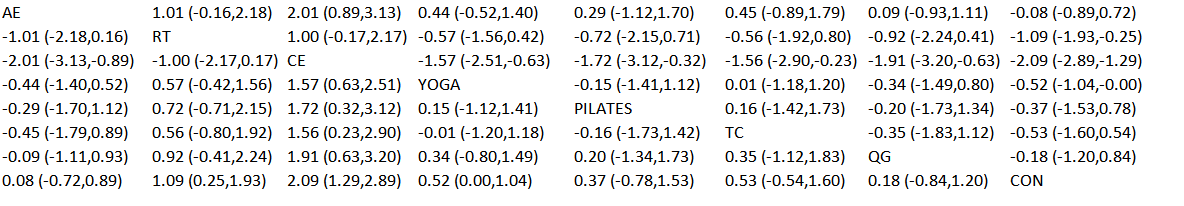
Table 11 Ranking of quality of life for different types of exercise less than eight weeks.


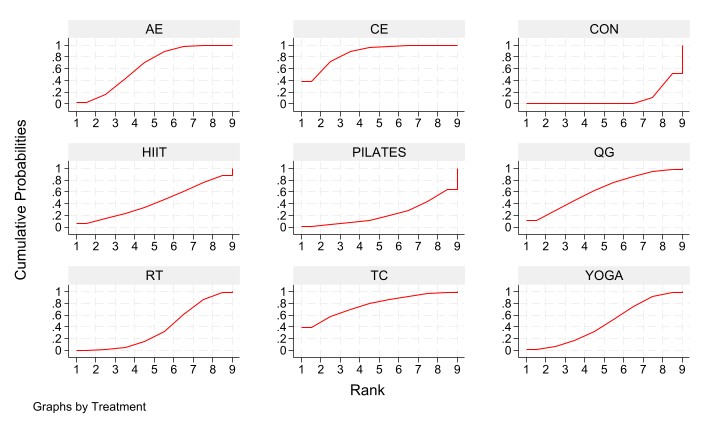


Figure 13 Cumulative ranking probability graph of quality of life after different exercise interventions more than eight weeks.


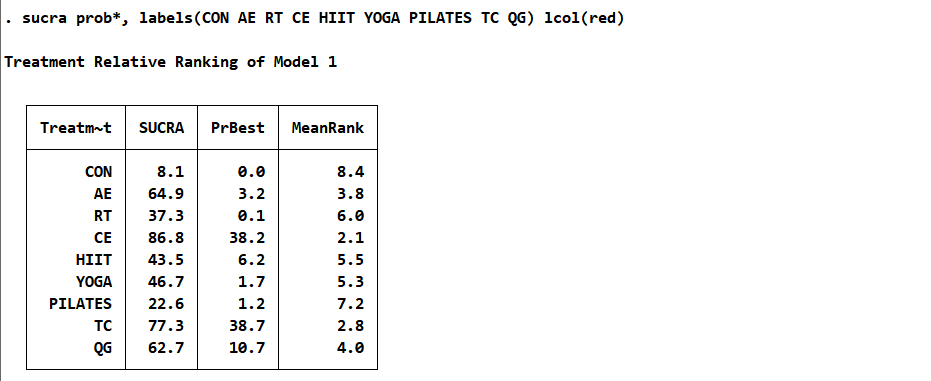


Figure 14 Ranking of quality of life for different types of exercise more than eight weeks.


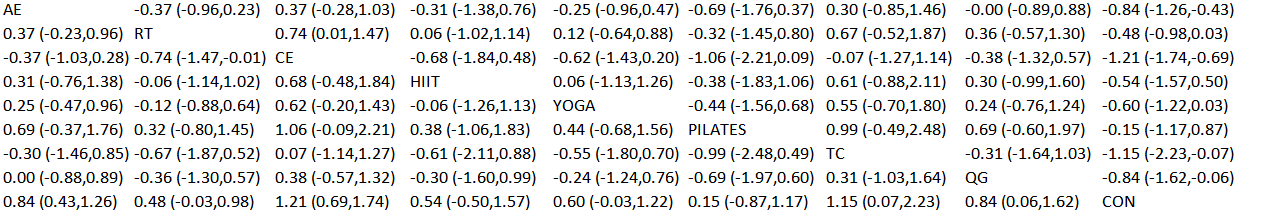


Table 12 Ranking of quality of life for different types of exercise more than eight weeks.


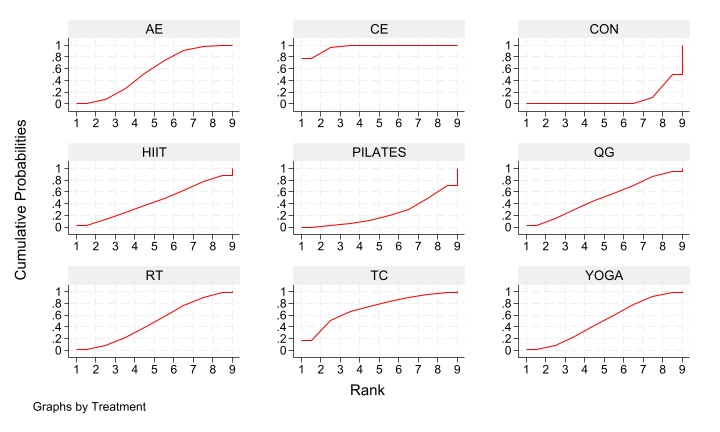


Figure 15 Cumulative probability plot of quality of life rankings after different exercise interventions (intervention frequency more than or equal to 3 times).


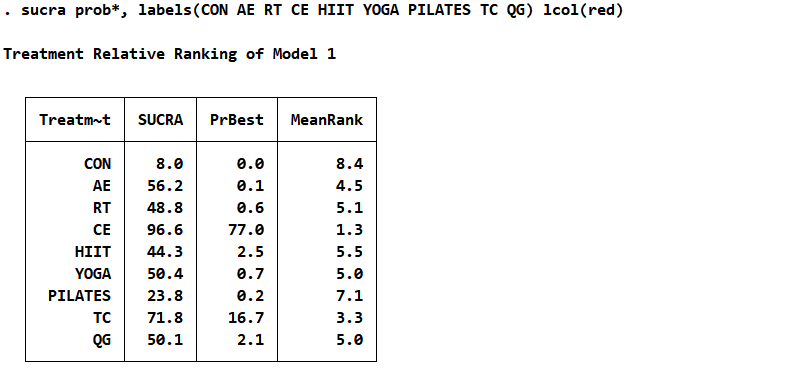


Figure 16 Ranking of quality of life for different types of exercise (intervention frequency more than or equal to 3 times).


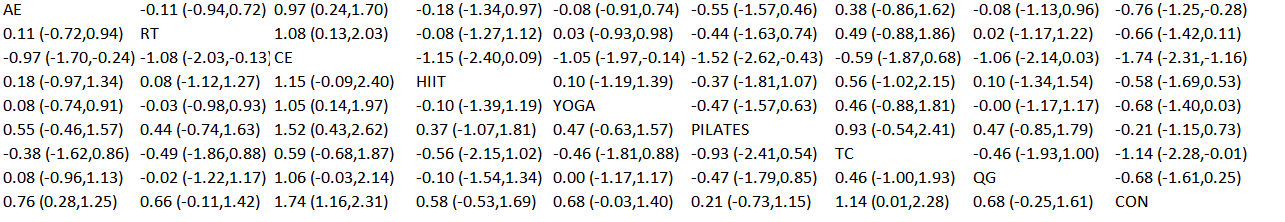


Table 13 Ranking of quality of life for different types of exercise (intervention frequency more than or equal to 3 times).


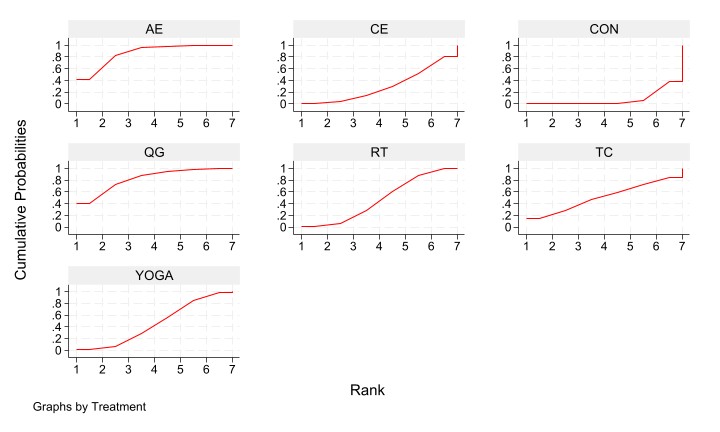


Figure 16 Cumulative probability plot of quality of life rankings after different exercise interventions (intervention frequency less than 3 times).


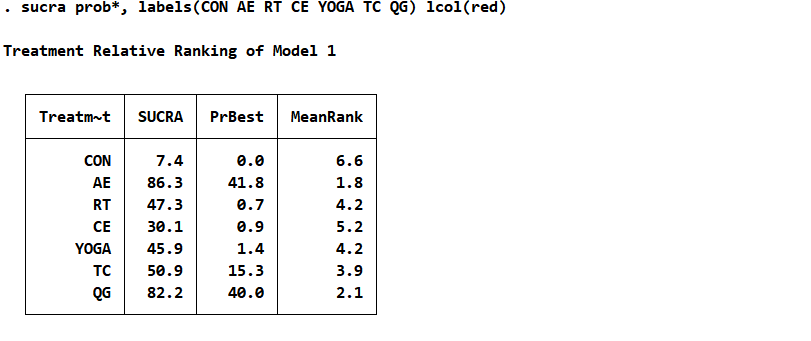


Figure 17 Ranking of quality of life for different types of exercise (intervention frequency less than 3 times).


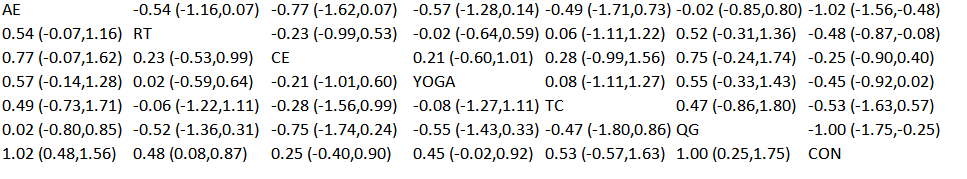


Table 14 Ranking of quality of life for different types of exercise (intervention frequency less than 3 times).


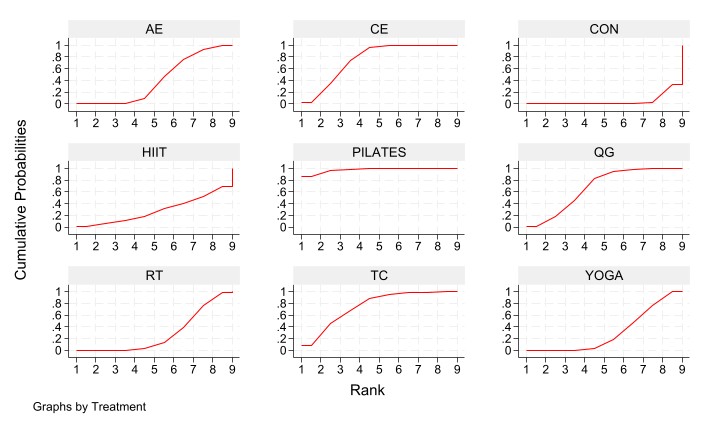


Figure 18 Cumulative probability plot of quality of life rankings after different exercise interventions (duration more than 40 minutes).


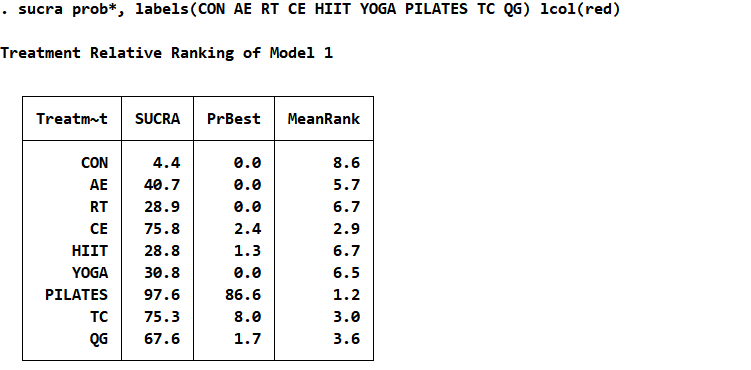


Figure 19 Ranking of quality of life for different types of exercise (duration more than 40 minutes).


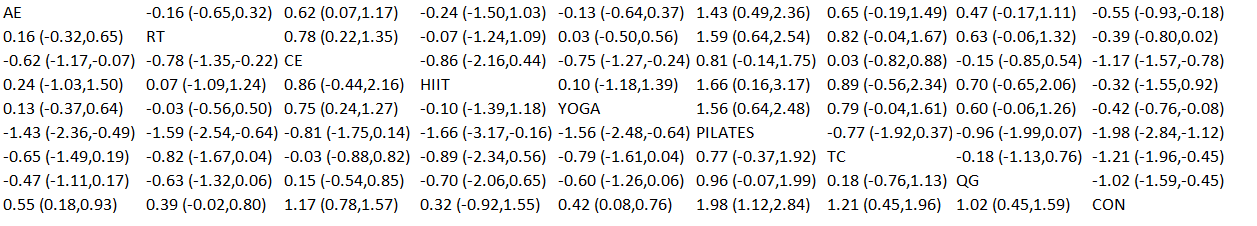


Table 15 Ranking of quality of life for different types of exercise (duration more than 40 minutes).


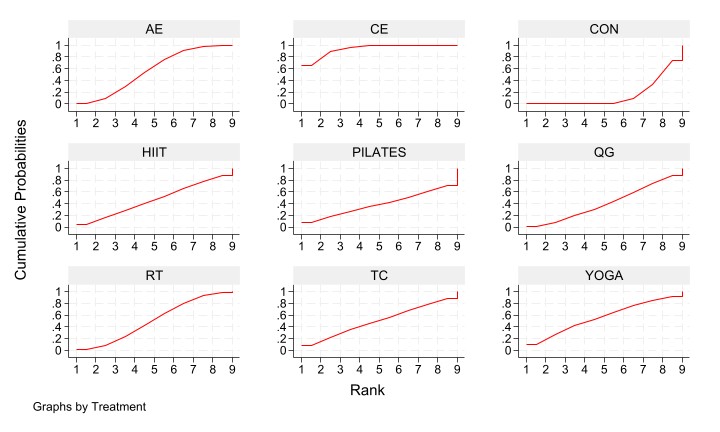


Figure 18 Cumulative probability plot of quality of life rankings after different exercise interventions (duration less than or equal to 40 minutes).


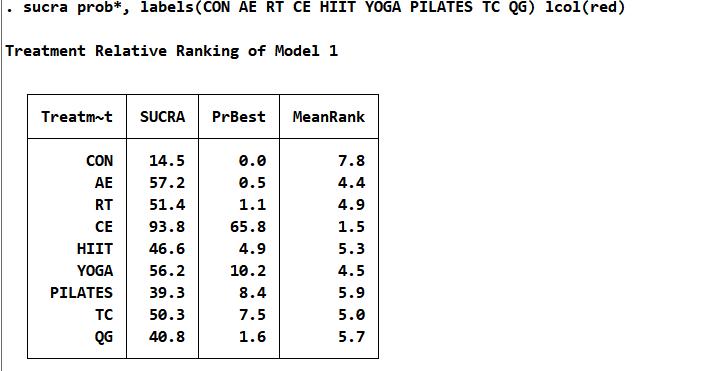


Figure 19 Ranking of quality of life for different types of exercise (duration less than or equal to 40 minutes).


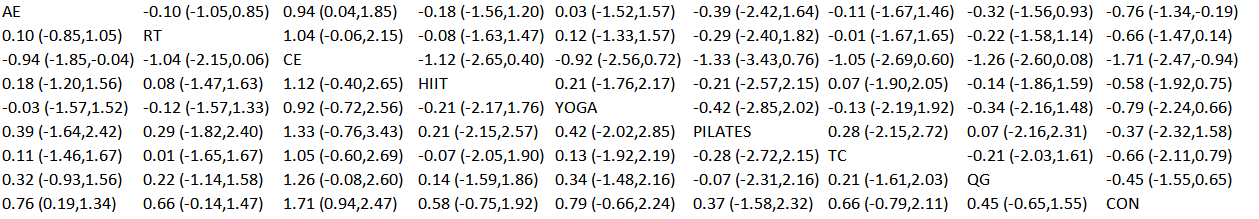


Table 15 Ranking of quality of life for different types of exercise (duration less than or equal to 40 minutes).

## 8.2 subgroup analysis for combined exercise for quality of life


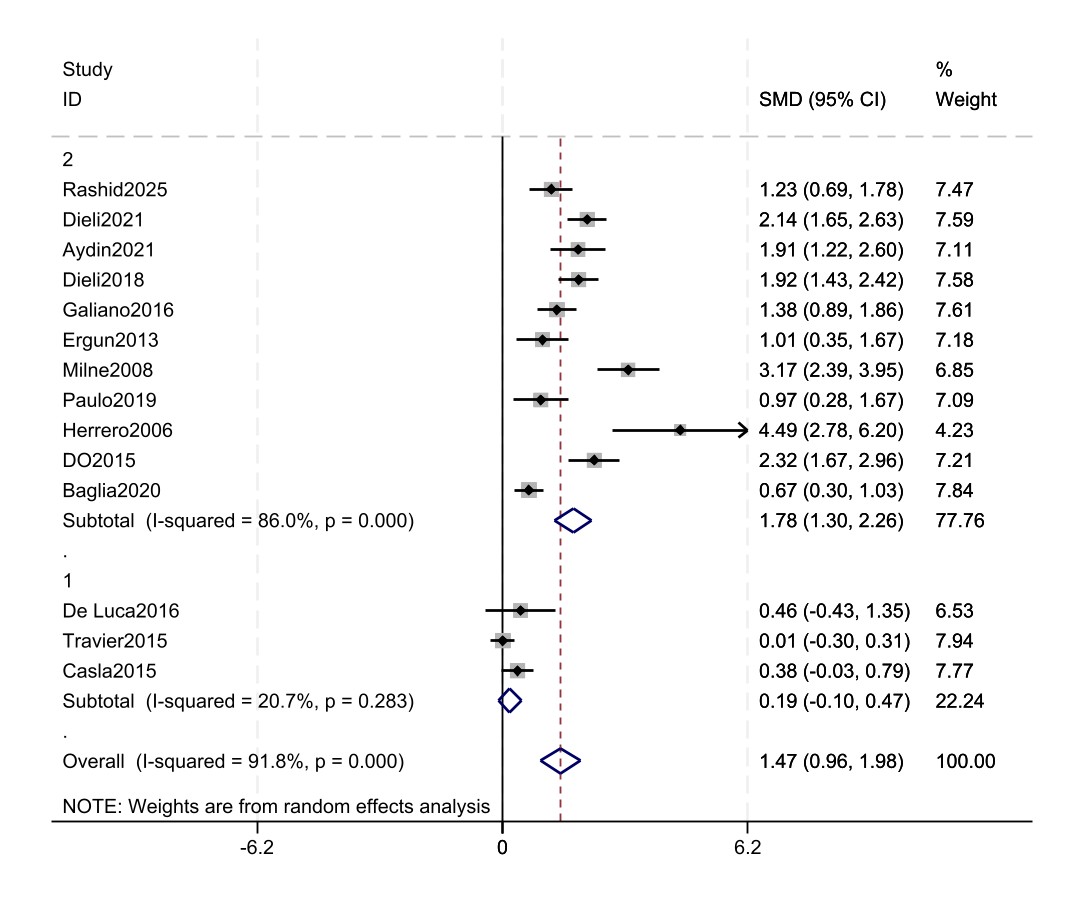


Figure 20 subgroup analysis for combined exercise for quality of life with different parameter(Frequency)

Note:1:Low,<3 times/week;2:High,≥3 times/week.


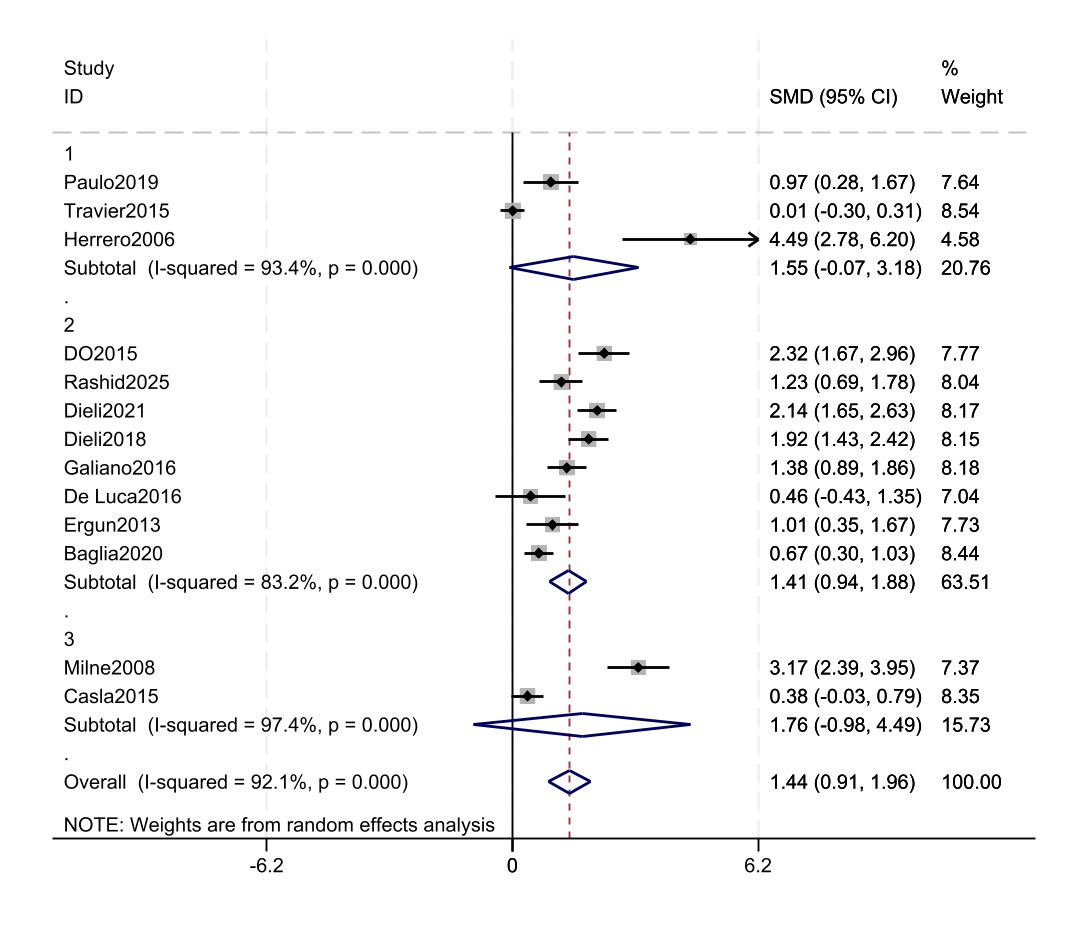


Figure 21 subgroup analysis for combined exercise for quality of life with different parameter(Duration)

Note:1:Short,≤40 minutes;2:Long,＞40 minutes;3:unclear.


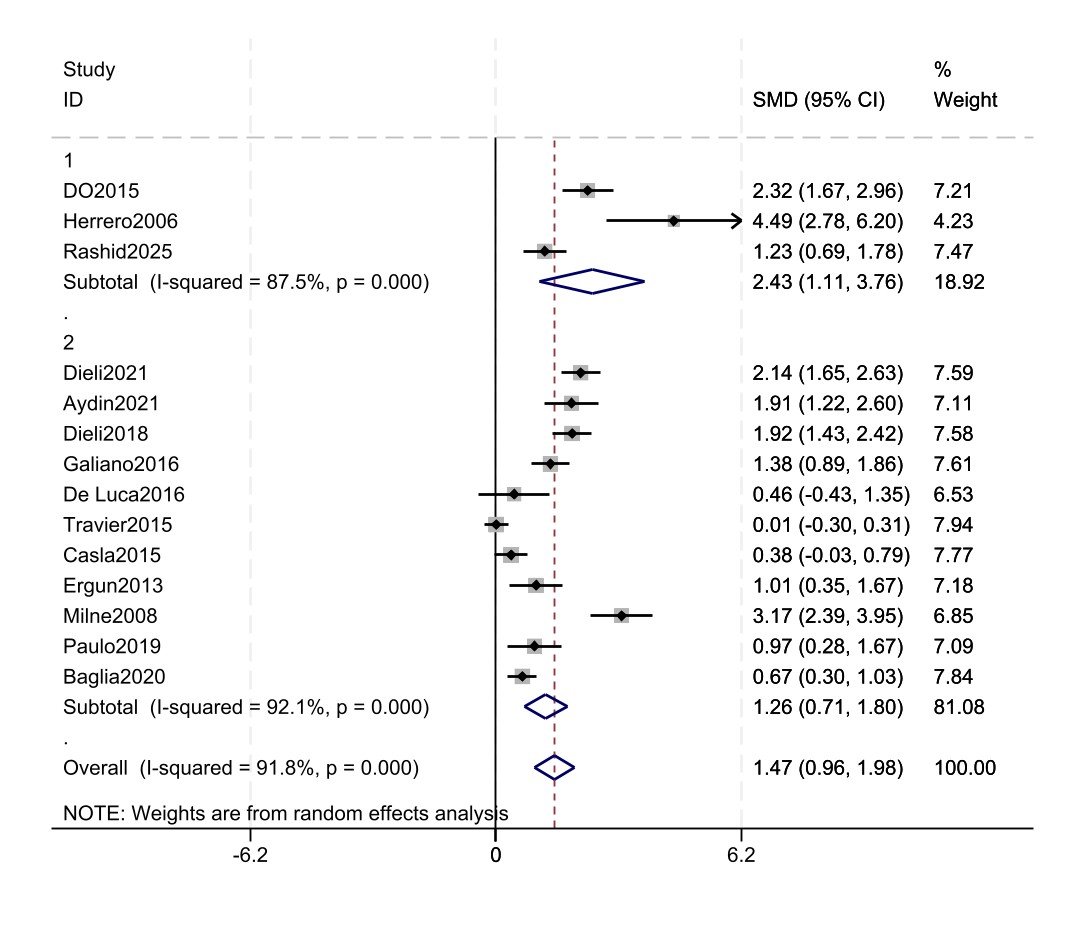


Figure 22 subgroup analysis for combined exercise for quality of life with different parameter(length)

Note:1:Short,≤8 weeks;2:Long,＞8 weeks.


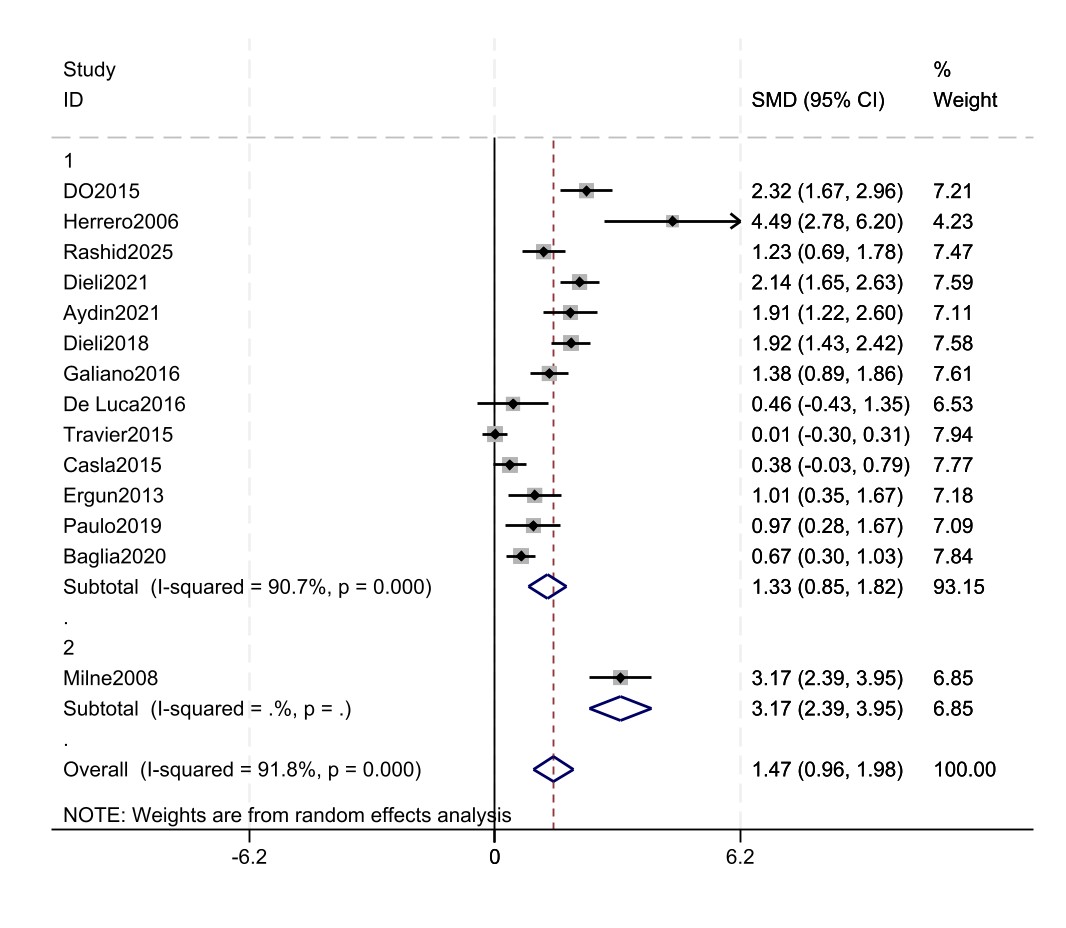


Figure 23 subgroup analysis for combined exercise for quality of life with different parameter(intensity)

Note:1:Moderate;2:unclear

# Appendix 9 The results of the sensitivity analysis

**9.1 Sensitivity analysis of quality of life**


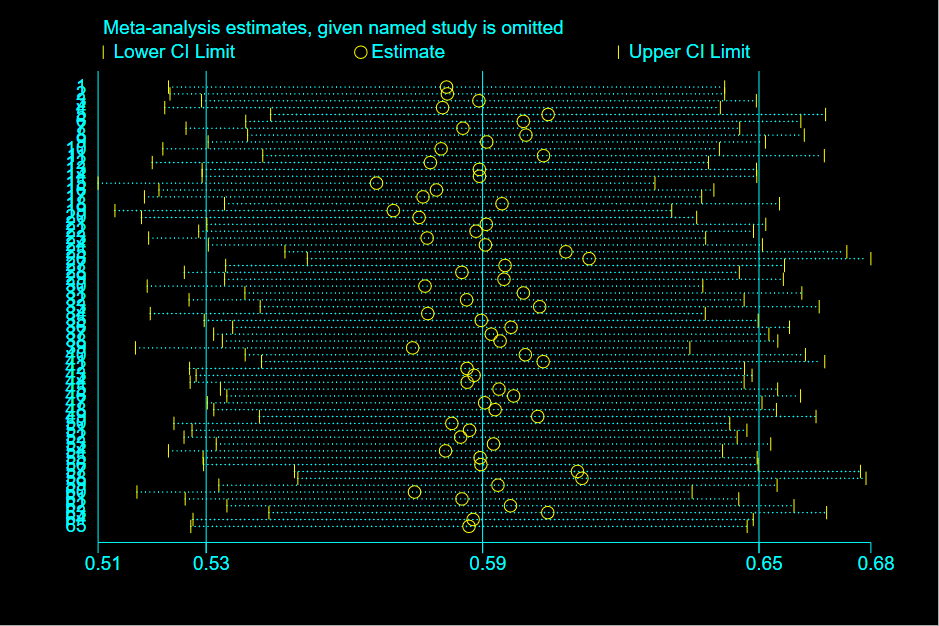


Figure 23 The result of sensitivity analysis of quality of life
